# ErbB2 Targeted Epigenetic Modulation: Anti-tumor Efficacy of the ADC Trastuzumab-HDACi ST8176AA1

**DOI:** 10.3389/fonc.2019.01534

**Published:** 2020-01-23

**Authors:** Ferdinando Maria Milazzo, Loredana Vesci, Anna Maria Anastasi, Caterina Chiapparino, Antonio Rosi, Giuseppe Giannini, Maurizio Taddei, Elena Cini, Valentina Faltoni, Elena Petricci, Gianfranco Battistuzzi, Laura Salvini, Valeria Carollo, Rita De Santis

**Affiliations:** ^1^Biotechnology R&D, Alfasigma SpA, Rome, Italy; ^2^Dipartimento di Biotecnologia, Chimica e Farmacia, Università degli Studi di Siena, Siena, Italy; ^3^Lead Discovery Siena srl, Siena, Italy; ^4^Fondazione Toscana Life Sciences, Siena, Italy; ^5^Histo-Cyto Service srl, Rome, Italy

**Keywords:** ADC (antibody drug conjugate), HDACi (Histone deacetylase inhibitor), trastuzumab, ErbB2, solid tumors

## Abstract

Targeted therapy using monoclonal antibodies conjugated to toxins is gaining space in the treatment of cancer. Here, we report the anti-tumor effect of a new antibody drug conjugate (ADC) delivering a HDAC inhibitor to ErbB2+ solid tumors. Trastuzumab was partially reduced with tris [2-carboxyethyl] phosphine (TCEP) and conjugated to ST7464AA1, the active form of the prodrug HDAC inhibitor ST7612AA1, through a maleimide-thiol linker to obtain the Antibody Drug Conjugate (ADC) ST8176AA1. The average drug/antibody ratio (DAR) was 4.5 as measured by hydrophobic interaction chromatography (HIC). Binding of ST8176AA1 to ErbB2 receptor and internalization in tumor cells were investigated by enzyme-linked immunosorbent assay (ELISA), surface plasmon resonance (SPR), cytofluorimetry, and High Content Screening (HCS) Imaging. The biological activity of the ADC was evaluated *in vitro* and *in vivo* by measuring cell proliferation/cell cycle, apoptosis/DNA damage, tubulin, and histone acetylation and modulation of Epithelial/Mesenchymal Transition (EMT) markers. Receptor binding and internalization of ST8176AA1 were confirmed to be similar to trastuzumab. Higher anti-tumor activity of ST8176AA1 compared to trastuzumab was observed *in vitro* in tumor cell lines. Such higher activity correlated with increased acetylation of histones and alfa-tubulin as a consequence of HDAC inhibitor-mediated epigenetic modulation that also induced increased expression of ErbB2 and estrogen receptor in triple negative breast cancer cells. Consistently with *in vitro* data, ST8176AA1 exhibited higher tumor growth inhibition than trastuzumab in xenograft models of ovary and colon carcinoma and in two patient-derived xenograft (PDX) models of pancreatic carcinoma. Immunohistochemistry analysis of tumor masses showed lower expression of the proliferation marker Ki67 and higher expression of cleaved caspase-3 in mice treated with the ADC compared to those treated with trastuzumab and results correlated with increased acetylation of both histones and tubulin. Collectively, present data indicate that ADC ST8176AA1 can target epigenetic modulation to ErbB2+ tumors. Interestingly, the amount of HDACi estimated to be delivered at the ST8176AA1 effective dose would correspond to ~1/1,000 of ST7612AA1 effective dose. Therefore, ST8176AA1 is an attractive new therapeutic candidate because it exhibits increased anti-tumor potency compared to trastuzumab by exerting epigenetic modulation at a much safer dose compared to standard HDACi-based therapeutic protocols.

## Introduction

Monoclonal antibodies (mAbs) targeting receptors of the Epidermal Growth Factor Receptor (EGFR) family, including anti-EGFR cetuximab and panitumumab and anti-ErbB2 trastuzumab and pertuzumab, are largely employed for cancer therapy in combination with other therapeutics. Antibodies contributed to improve clinical responses, patient quality of life and survival. Nevertheless, there is a growing awareness that in many cases antibody therapies are far from optimal, primarily because, like chemotherapeutics, they induce tumor cell resistance to inhibitory activities. Therefore, there is a need to increase the number of therapeutic antibodies (1) and there is a trend to harness them with additional anti-tumor features. The latter translated into clinical and commercial success of Antibody Drug Conjugates (ADCs) including emtuzumab ozogamicin (Mylotarg; Pfizer/Wyeth), ibrentuximab vedotin (Adcetris; Seattle Genetics), ado-trastuzumab emtansine (Kadcyla; Roche), inotuzumab ozogamicin (Besponsa; Pfizer) (2) and recently approved recombinant immunotoxin moxetumomab pasudotox-tdfk (Lumoxiti; Astra Zeneca) (3) and polatuzumab vedotin-piiq (Polivy; Roche) (4). It is to note that trastuzumab-emtansine (T-DM1, Kadcyla) is the only one product approved for the treatment of solid tumors. Trastuzumab is a humanized monoclonal antibody directed to ErbB2 growth factor receptor. It was first approved by FDA in 1998 and ever since employed widely worldwide for the treatment of primary and metastatic ErbB2+ breast and gastric cancer. Trastuzumab changed the treatment paradigm of breast cancer but it did not solve the problem of tumor resistance that, in turn, promoted its use in combination with chemo or with the second anti-ErbB2 antibody pertuzumab (5). Recently, trastuzumab was harnessed with highly cytotoxic properties by conjugating it to the cytoskeleton disrupting agent emtansine obtaining the ADC T-DM1. This product, as any other anti-cancer treatment, is also facing intrinsic and extrinsic resistance mechanisms (6) and significant side effects the latter related to the payload off-target toxicity (7). Overall, there is still a wide space for investigational activities aiming at improving the properties of trastuzumab.

Because epigenetic abnormalities are involved in the hallmarks of cancer, histone deacetylase inhibitors (HDACis) have recently shown efficacy against both hematological and solid cancers. In particular, in cancer, decreased histone acetylation, as consequence of HDAC hyperactivity, leads to the repression of genes resulting in uncontrolled cell proliferation and malignancy. Moreover, during cancer progression, aberrant activity of HDACs leads to loss of cell adhesion, resulting in cell migration, invasion, and angiogenesis (8). For these reasons, several HDACi drug candidates are being investigated and four have been approved for hematologic malignancies. Further strategies are being carried out to achieve dual molecular targeting, such as chimeric compounds that combine HDACi and tyrosine kinase inhibitors (TKi) moieties (9), as well as compounds acting simultaneously against Estrogen Receptors (ER) and HDACs, with the aim to modulate multiple cellular pathways and obtain higher efficacy compared to that of single-target drugs (10).

We recently described a new class of ADCs where the antibody cetuximab was charged with the low toxic HDAC inhibitor ST7612AA1 (11). Such ADCs, coded ST8154AA1 and ST8155AA1, are the first example of products targeting epigenetic modulation to tumors. ST7612AA1 is a pan-HDACi previously shown to exhibit anti-tumor activity against a variety of tumor models (12–14). Despite efficacy, translation of ST7612AA1 into human therapeutic protocols is hampered by the risk of systemic side effects that are also observed with the previously approved HDACi vorinostat (15). To be effective, this class of molecules must be administered daily at high dose thus exerting systemic epigenetic modulation ultimately leading to tolerability issues. Therefore, there is a need to improve tumor targeting of HDACi. We previously demonstrated that it is possible to use the anti-tumor antibody cetuximab to deliver an HDACi to EGFR+ tumors and these ADCs exhibited a better efficacy/toxicity ratio than either cetuximab or HDACi at their respective optimal doses (11).

In the present work, we describe, for the first time, the generation and characterization of new ADCs, coded ST8176AA1 and ST8178AA1, where the active form of HDACi ST7612AA1 is linked to trastuzumab. The present work demonstrates in five animal studies, including two patient-derived pancreatic models, that the anti-tumor activity of trastuzumab can be improved by the conjugation to a safe and otherwise inactive dose of an epigenetic modulator moiety.

## Materials and Methods

### Synthesis and Characterization of ADCs

The chemical synthesis of the payloads was previously described (11). The scheme of conjugation of payloads to trastuzumab and the analytical profiles of the ADCs are shown in the Supplementary Figures S1–S5.

In brief, the ADC ST8176AA1 was obtained by a two-step reaction. First, trastuzumab in PBS, pH 7.4, was partially reduced with 1 nM TCEP at 37°C for 2 h, then the payload (20 mM in DMSO) was added to the reaction and the solution was left under shaking for 1 h at room temperature. The product was dialyzed to remove unreacted payload and analyzed by Hydrophobic Interaction Chromatography (HIC).

The mobile phase A was 1.5 M ammonium sulfate, 50 mM sodium phosphate dibasic dihydrate pH 7.0/isopropanol (95:5 v/v). The mobile phase B was 50 mM sodium phosphate pH 7.0/isopropanol (80:20 v/v). The analysis by HIC was performed on an UHPLC Ultimate 3000 (Dionex, now Thermo Fisher) using a column MabPac HIC-Butyl 100 × 4.6 mm, 5 μm (Thermo Fisher). The ADC ST8178AA1 was obtained by adding the payload to the antibody at 1:20 molar ratio. The resulting solution was left under shaking for 1 h at room temperature. The product was dialyzed to remove unreacted payload and the DAR was determined by MALDI mass spectrometry. Briefly, 1 μL of desalted trastuzumab or ADC ST8178AA1 was mixed with 1 μL of a saturated solution of s-DHB in 0.1% TFA in acetonitrile:water (50:50, v/v). The resulting mixture was spotted on the MALDI target and it was left to dry in the air. Then 1 μL of the matrix solution was added to each spot and it was left to dry again.

The mass spectra were acquired using an Ultraflex III (Bruker Daltonics, GmbH) in linear mode. A protein standard II mixture, from Bruker Daltonics, was used for calibration. The mass spectra were acquired over the mass range m/z 50–180 kDa.

The DAR was calculated from the difference in molecular weight between the ADC and native trastuzumab. The gradient started with 85% of A and 15% B and it was hold constant for 1 min after injection. Then mobile phase B was increased at 100% in 25 min. The flow rate was 500 μL/min, the column was maintained at 27°C and 20 μL of each sample were injected into the column. Detection was carried out at 280 and 220 nm.

In the case of ADC ST8176AA1, the average DAR was calculated considering the area of the peaks shown in the chromatograms using the following formula:

$$\begin{array}{l} \bar{DAR}=\frac{\overset{8}{\underset{0}{\sum }}nA_{DAR_{n}}}{\overset{8}{\underset{0}{\sum }}A_{DAR_{n}}} \end{array}$$

### ErbB2 Binding by Surface Plasmonic Resonance (SPR)

Binding kinetic constants by SPR were determined by using a Biacore T200 instrument (GE Healthcare). Recombinant human ErbB2-HisTag (Sino Biological) at 1.67 μg/mL, in acetate buffer pH 5, was immobilized at 42 RU on the surface of flow cell 2 of a sensor chip CM5 by classical amine coupling procedure, while surface in flow cell 1 was blank immobilized to be used as control. Test items were flowed at 15 μL/min through flow cell 1 and 2 at 0.41, 1.23, 3.7, 11.1, 33.3, 100, and 300 nM concentrations in HBS-EP+ buffer (GE Healthcare) for a contact time of 480 s. After a dissociation time of 1,200 s, both flow cells surfaces were regenerated by flowing 10 mM glycine pH 1.5 at 15 μL/min, for 45 s. Analyses were performed in triplicate and sensorgrams were obtained by subtraction of flow cell 1 curves from flow cell 2 curves. Kinetic constants were obtained using 1:1 binding model by BIA evaluation 3.0 software. Data reported as mean of three values ± standard deviation (SD).

### Cell Lines

The following human carcinoma cell lines were used: ovary (SKOV-3 and IGROV-1 from Istituto Nazionale Tumori Milano, and Caov3 from DSMZ); colon (LS174T and HCT-116 from ATCC); non-small cell lung carcinoma (A549 from DSMZ, and NCI-H1975 from ATCC); pancreas (MiaPaCa2 from DSMZ, and Capan-1 from ATCC); stomach (N87 from ATCC), breast (SKBR3 from Istituto Superiore di Sanità, MCF-7 from ATCC, BT549 and MDA-MB231 from DSMZ). SKOV-3, IGROV-1, HCT-116, N87, SKBR-3, NCI-H1975 and Capan-1 cells were cultivated in RPMI-1640 supplemented with 10% FBS, 2 mM L-glutamine and 1% non- essential amino acids (NEAA); LS174T cells were cultivated in EMEM supplemented with 10% FBS, 2 mM L-glutamine and 1% NEAA; Caov-3, A549, MiaPaCa2, MCF-7, MDA-MB231 and BT-549 cells were cultivated in DMEM supplemented with 10% FBS and 2 mM L-glutamine. 32D, 32D_B2/B3 and 32_EGFR cells (16) (kindly provided by Prof Maurizio Alimandi, University of Rome La Sapienza, Italy) were routinely cultivated in RPMI supplemented with 10% FBS, 1 ng/mL interleukin-3 (Cell Signaling).

All experiments were performed starting from frozen cell stocks of working cell banks within 6–8 passages after thawing.

### Cell Proliferation

The effect of test items on cell proliferation was evaluated on human ovarian carcinoma (SKOV-3, IGROV-1 and Caov-3) or colon carcinoma (HCT-116 and LS174T) cell lines. Cells were seeded at 3,000–5,000 cells/well into 96-well plates in complete culture medium and incubated 6 days, in quadruplicate, with scalar concentrations of test items ranging from 500 to 6.25 nM. Inhibition of cell proliferation was measured by CellTiter-Glo Luminescent Cell Viability Assay (Promega), by using Veritas luminometer (Promega). Data were expressed as the average inhibition percentage (± SD) of at least two independent experiments. In some experiments, cell proliferation was assessed counting cells by NucleoCounter NC200 (ChemoMetec) and then calculating the cellular doubling time.

Murine cells transfected for overexpressing the human receptors ErbB2/ErbB3 (32D_B2/B3) or EGFR (32D_EGFR), and their untransfected counterpart (32D) were washed with serum-and IL3-free medium and starved 2 h. After washing twice with PBS, cells were seeded in 96-well plates in RPMI 1% FBS and incubated 2 h with scalar concentrations of test items, before adding 1.5 ng/mL recombinant human NRG-b1/HRG1-β (R&D Systems) or 0.5 ng/mL IL-3, for 32D_B2/B3 and 32D cells, respectively. After 48 h, cell growth was measured by CellTiter-Glo, as above.

### Cytofluorimetry Analysis

For testing binding, pellets of different tumor cell lines were incubated 1 h at 4°C with test items at 5 μg/mL in 100 μL. After washings, cells were incubated with mouse anti-human PE- or FITC-conjugated Ig (Becton-Dickinson). Cytofluorimetry analysis was performed by using FACSCalibur (Becton-Dickinson).

For cell cycle experiments, LS174T cells were incubated up to 144 h with scalar concentrations of test items at concentrations ranging from 500 to 125 nM. At selected time points, cells were detached by Trypsin/EDTA and counted by using NucleoCounter NC200 (ChemoMetec). For DNA analysis, cellular nuclei stored at −20°C in 70% (v/v) ethanol were washed and resuspended in 100 μL PBS and then stained, for 30 min at room temperature, with 100 μL of a solution containing 0.1 % (w/v) sodium citrate, 100 μg/mL RNase, 0.1% Triton-x 100 and 50 μg/mL propidium iodide (Sigma-Aldrich). Cell cycle and apoptosis analyses were performed by using FACSCanto II (Becton-Dickinson). Data elaborated by ModFit LT v3.0 software (Verity Software House).

### High Content Screening (HCS) Fluorescence Imaging

Cells were seeded into 96-well microtiter plates in complete medium and then incubated with test items for the indicated times. After cell fixation with 4% formaldehyde in PBS, permeabilization with 0.2% Tween-20 in PBS (PBS-T) and blocking with 2% BSA in PBS-T, test items were detected by FITC- or PE-conjugated mouse anti-human Ig (Becton-Dickinson). Expression of protein targets after cell fixation, permeabilization and blocking as described above, was evaluated by adding the following specific primary antibodies: rabbit anti-phospho-HER2 (Tyr1221/1222) (Abnova); rabbit anti-phospho-Akt (Ser473), -acetyl-Histone H3 (Lys9/Lys14), -P53 (7F5), -Bcl-XL, -cleaved caspase-3, -cleaved PARP (Asp214) (D64E10), -E-cadherin (24E10) and -ERalpha (D6R2W) (Cell Signaling); rabbit anti-phospho-Erk, -acetyl-Histone H4 (Ser1/Lys5/Lys8/Lys12) and -P21 (Santa Cruz); rabbit anti-Bcl-2 and -Claudin 2 (Abcam); rabbit anti-HER2/ErbB2 (Sigma-Aldrich); mouse anti acetyl-tubulin (6-11B-1), -Vimentin (RV202) and -N-cadherin (D-4) (Santa Cruz); mouse anti-HSP70 (BRM-22) (Sigma-Aldrich). FITC-conjugated goat anti-rabbit or goat anti-mouse IgG (Becton-Dickinson) were then added, according to the primary antibody used. Cells were counterstained with Draq5 dye (Cell Signaling). Fluorescence signals were acquired by the High Content Screening (HCS) system Operetta (Perkin Elmer).

### Western Blotting

Tumor cells were seeded in 10-cm culture plates in complete medium and then incubated with 10–40 μg/mL test items, for 3 h. Cells were then washed twice and whole cell lysates prepared by incubation, 10 min on ice, with 1× Lysis Buffer (Cell Signaling) supplemented with protease inhibitors. Cell lysates were subjected to sonication prior to centrifugation at 14,000 × g, for 10 min at 4°C, to remove cell debris. Protein content was determined by Bradford method. Equal amounts of soluble proteins were separated by SDS-PAGE and then transferred to nitrocellulose membrane (Amersham Hybond-ECL) (GE Healthcare). Membranes were blocked 3 h at room temperature with 5% non-fat dry milk in PBS 0.05% Tween-20 (PBS-T) before overnight incubation, at 4°C, with one of the following primary antibodies: rabbit anti-acetyl Histone H4 [Ser1/Lys5/Lys8/Lys12] (Santa Cruz) or mouse anti acetyl-tubulin (6-11B-1) (Santa Cruz). Anti-β-actin antibody (Sigma-Aldrich) was used to normalize protein loading. After washings with PBS-T, membranes were incubated 1 h with the appropriate secondary HRP-conjugated anti-rabbit or anti-mouse IgGs (Sigma-Aldrich and Amersham GE-Healthcare, respectively). Immunoreactive bands were visualized by enhanced chemiluminescence phosphoimaging (STORM, Molecular Dynamics) or by exposure to X-ray film (Amersham Hyperfilm ECL) (GE Healthcare).

### Real-Time Quantitative Polimerase Chain Reaction (qPCR)

Total RNA was extracted by using Purelink RNA Mini kit (Invitrogen, Paisley, UK) from LS174T cells, upon 6 h treatment with test items (100 nM ST7612AA1, 250 nM ST8176AA1 or trastuzumab, or 10 μM GANT58, a reference Hh inhibitor) in serum-free medium (Sigma- Aldrich), followed by incubation for additional 18 h with 100 nM Hh agonist SAG (Sigma-Aldrich). Cells only grown in serum-free medium were used as unstimulated (reference control) cells. RNA was then retrotranscribed using the SuperScript IV VILO Mastermix (Invitrogen, Paisley, UK), according to the manufacturer's instructions. Real time quantitative PCR analysis was performed using the Luna® Universal Probe qPCR Master Mix (New England Biolabs, Ipswich, MA, USA) and the following TaqMan Gene Expression Assays (Applied biosystems, Foster City, CA): Hs00171790_m1 [GLI-1], Hs01119974_m1 [GLI-2], Hs00181117_m1 [PTCH1], Hs00170665_m1 [SMO], Hs00960520_m1 [SUFU] and Hs00939627_m1 [for the housekeeping gene GUSB]. The 7900HT Sequence Detection System instrument and software (Applied Biosystems) were used to quantify the mRNA levels of the target genes, according to a six-point serial standard curve generated for each gene. Results were ultimately expressed, after normalization to the housekeeping gene GUSB, as relative expression as compared to not stimulated cells.

### ELISA

Immunoreactivity of ST8176AA1 or trastuzumab for human ErbB2 receptor was assessed by antigen-specific ELISA. Briefly, Immuno™ MAXISORP 96-well plates (Nunc) were coated at 4°C overnight with 100 μL/well of recombinant human Erb2/HER2 Fc chimera (Sino biological) at 2 μg/mL. Plates were washed with PBS, 0.1% Tween 20 and blocked with 300 μL/well of PBS, 0.1% Tween20, 1% BSA for 2 h at room temperature. After three washings, plates were incubated with serial dilution of test items for 1 h at room temperature. Plates were then washed three times and incubated with HRP-conjugated rabbit anti-human K light chain antibody (Sigma-Aldrich) diluted 1:10,000 in blocking solution, 1 h at room temperature. After three washings, 200 μL of TMB substrate (Sigma-Aldrich) were added to each well and incubated for 30 min at 37°C. The reaction was blocked with 100 μL of 0.5 M H_2_SO_4_ and plates read at 450 nm by using ELISA spectrophotometer Sunrise (TECAN).

### Animal Studies

#### Tumor Xenograft Models

Animal studies were performed in accordance with the “Directive 2010/63/UE” on the protection of animals used for scientific purposes, made effective in Italy by the Legislative Decree 4 March 2014, n. 26, and ARRIVE guidelines (17). At the end of the treatment period and before necropsy, mice were euthanized by CO_2_ asphyxia as indicated in the AVMA (American Veterinary Medical Association) panel on euthanasia and according to the guidelines described in UKCCR, United Kingdom Co-ordinating Committee on Cancer Research, 1998. Pathogen-free female nude mice weighing 20 ± 2 g, aged 4–5 weeks (Envigo) were used for each study. All animal procedures were approved by the Animal Housing of Plaisant/Takis. Animals were acclimated for 1 week before initiation of the studies. The daily light cycle extended from 7 a.m. to 7 p.m. and room temperature was maintained at 22.0 ± 2.0°C. Throughout the study, mice were allowed to consume food and water *ad libitum*.

Following acclimatization, 5 × 10^6^ SKOV3 ovary carcinoma or LS174T colon carcinoma cells were subcutaneously injected, in 100 μL, into the right flank of the mice or, alternatively, 10 × 10^6^ cells resuspended in 200 μL PBS containing 20% matrigel were intraperitoneally injected for establishing an orthotopic model.

When subcutaneous tumor lesions reached a mass of about 50 mm^3^, the animals were randomly assigned to groups (*n* = 10–12/group) and treated i.p. with PBS (control) or with 4 doses of 15 or 30 mg/kg ST8176AA1 or trastuzumab once every 4 days, starting 10 days after tumor cell transplantation. For the orthotopic tumor models, mice were treated (*n* = 10/group) intraperitoneally with ST8176AA1 or trastuzumab (4 doses of 15 mg/kg once every 4 days, starting 3 days after tumor cell transplantation). Control group received PBS.

#### Patient-Derived Tumors

Female NOD SCID mice (Jackson) of 20 ± 2 g were used. Animals were housed in individual HEPA ventilated cages (Innocage® IVC, Innovive USA). Fluorescent lighting was provided on a 12-h cycle. Temperature and humidity were monitored and recorded daily and maintained to the maximum extent possible between 20 and 23°C and 30–70% humidity, respectively. 2920X.10 18% soy irradiated rodent feed (Harlan) and autoclaved acidified water (pH 2.5–3.0) were provided *ad libitum*. Cells from PA5363 and PA5366 (77,000 and 56,000 cells/mouse, respectively, in 200 μL) from the bank cryovals of Crown Bioscence were washed in PBS and counted in cold PBS. Cell suspensions were mixed with an equal volume of Cultrex ECM and kept on ice to avoid the solidification of ECM. Vehicle and ST8176AA1 or trastuzumab (15 mg/kg) were intraperitoneally injected every 4 days for 5 times, starting 28 or 35 days after tumor transplantation for PA5363 and PA5366, respectively.

#### Evaluation of Tumor Growth and Survival Analysis

Tumor growth (length, width, and volume) was measured twice a week by using a digital caliper. Tumor volume was calculated by using the following formula: V = Length × Width2/2. All experimental animals were euthanized when the tumors reached a volume ≥ 1,200 mm^3^, as required by institutional guidelines.

Before sacrifice, mice received another dose of drugs and 24 h after were sacrificed by CO_2_. Immediately after euthanization, tumors were excised, weighed, snap-frozen in liquid nitrogen and stored at −80°C or fixed in 10% phosphate buffer formalin at +4°C.

In orthotopical mice models, survival data were plotted on Kaplan–Meier's survival curves using GraphPad Prism 5.02. Median survival time was also evaluated. The log-rank test was used to compare the survival rates between the 2 groups. A *P* < 0.05 was considered statistically significant.

#### Histology and Immunohistochemistry (IHC)

Tumor masses were harvested and fixed in 10% phosphate-buffered formalin at 4°C. Samples were then dehydrated in ascending concentrations of ethanol, cleared with xylene and paraffin embedded. Tissue slices were obtained using a rotary microtome (5 μm sections) and processed for histology and IHC. For histology, hematoxylin/eosin staining was performed according to standard methods.

For IHC, after deparaffination and rehydration, sections were treated with 10 mM citrate buffer and 0.05% Tween 20 pH 6.0 (Sigma-Aldrich) in a microwave for 15 min for antigen retrieval, followed by quenching of endogenous peroxidase activity with 3% H_2_O_2_ in PBS (v/v) for 5 min. Sections were then incubated overnight at 4°C, in a humidified chamber, with specific antibodies against Ki-67 (Novus Biologicals), cleaved caspase-3 (Cell Signaling), acetyl histone H3 (Cell Signaling), or acetylated-alpha-tubulin (Santa Cruz Biotechnology). Negative controls were incubated without primary antibodies under identical conditions. Sections were then incubated with the appropriate biotinylated secondary antibody (1:300), followed by conjugated horseradish peroxidase-streptavidin and 3,3′-diaminobenzidine (ABC kit) (Vector Laboratories). Counterstaining with hematoxylin. Images were captured using optical microscope Eclipse E800 (Nikon Corporation) equipped with a JVC KY-F55B color video digital camera. IHC staining was quantified as the number of positive cells × 100/total number of cells, in five fields from two serial sections/mouse. All tissue sections for IHC analysis were scored by two independent pathologists and subsequently data confirmed by computerized measurements (18). Data are presented as the mean ± standard error (SE) of 10–12 mice/group.

### Statistical Analysis

All statistical tests for *in vivo* and *ex vivo* experiments were done using the statistical package programme GraphPad Prism 5.02. All values were expressed as the mean ± SE or ± SD. Non-parametric analyses (Mann-Whitney's *U*-test) were used in the statistical computations for tumor growth and body weight. Differences were considered statistically significant when *P* < 0.05.

## Results

### Generation and Affinity Characterization of Trastuzumab-HDACi ADCs

**Figure 1:**
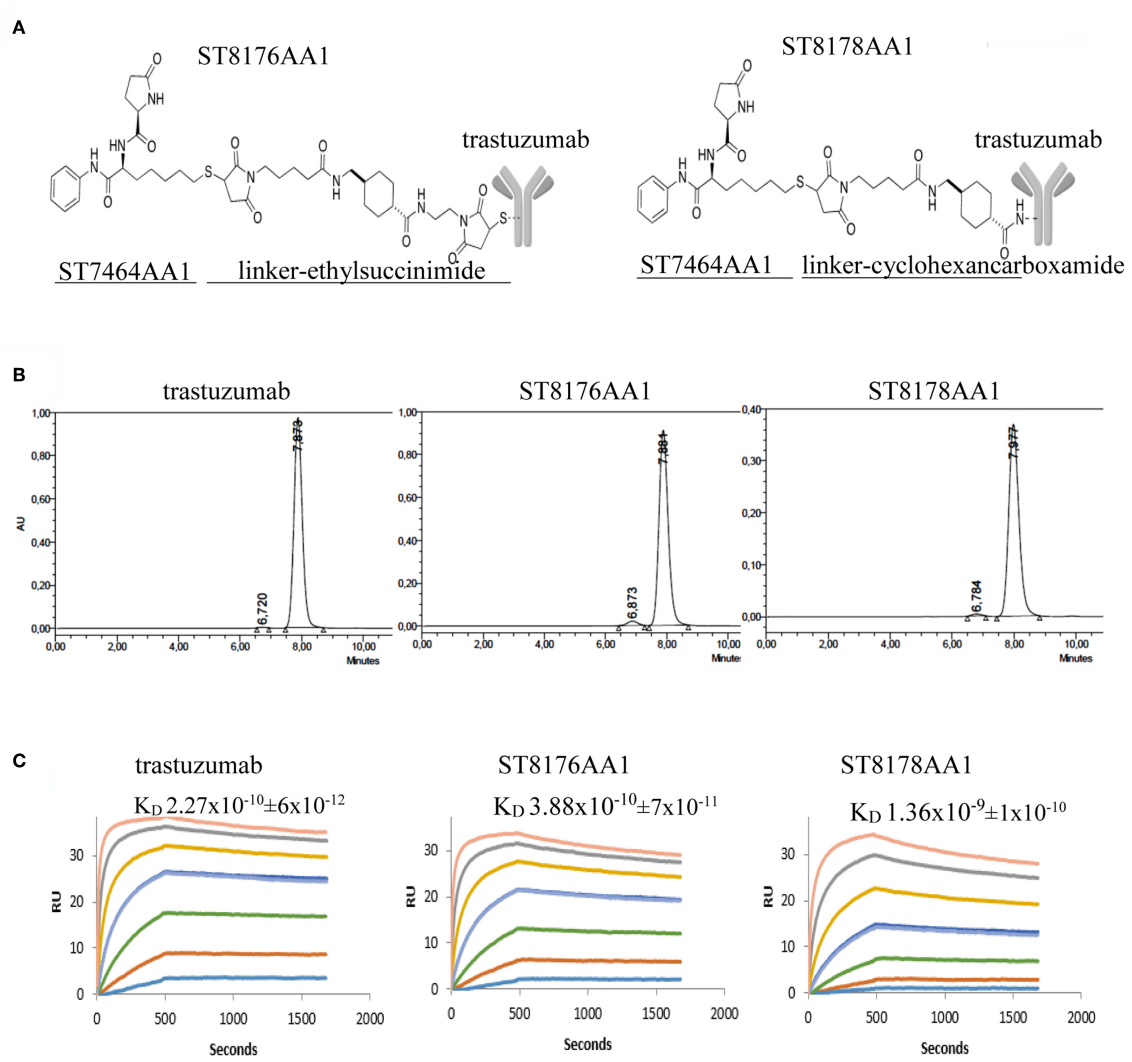
Generation and preliminary characterization of trastuzumab-HDACi ADCs. **(A)** Schematic representation of ADC ST8176AA1 and ST8178AA1. **(B)** Size exclusion chromatography. **(C)** ErbB2 binding affinity by Surface Plasmonic Resonance (SPR). Analysis on BiaCore T200. Test items were flowed at 0.41–300 nM concentrations. Sensorgrams fitting 1:1 model provided K_D_ values that are reported in each graphic as mean of three independent determinations ± SD. One representative sensorgram of each test item is shown.

With the intent to deliver epigenetic modulation to ErbB2-expressing tumors, we conjugated an HDACi-based payload to cysteines or lysines of trastuzumab (Figure 1A). ST8176AA1 and ST8178AA1 were obtained according to a conjugation protocol previously described (11) and briefly summarized in Supplementary Figure S1. The drug:antibody ratio (DAR) was calculated based on profiles of hydrophobic interaction chromatography (HIC) or MALDI for ST8176AA1 and ST8178AA1, respectively (Supplementary Figures S2–S5). Native mass spectra obtained for trastuzumab and ST8176AA1 are reported in Supplementary Figure S3 which, after deconvolution, showed as major component the species containing four payloads (Supplementary Figure S4).

Molecular integrity of both conjugates was confirmed by size exclusion chromatography that also showed the presence of some aggregates that were in any case <3% (Figure 1B). Such aggregates did not further increase upon storage of the ADCs at +4°C up to 24 months (data not shown). ADC binding affinity was tested by Surface Plasmonic Resonance in comparison with trastuzumab. Data in Figure 1C show that ST8176AA1 exhibited kinetic profiles more similar to trastuzumab than ST8178AA1, consistently with lower DAR (4.5 ± 0.5 vs. 8.0 ± 0.7, respectively, as an average of three distinct preparations). On this basis, ADC ST8176AA1 was selected for further characterization.

### Biochemical and Biological Characterization of ST8176AA1

**Figure 2:**
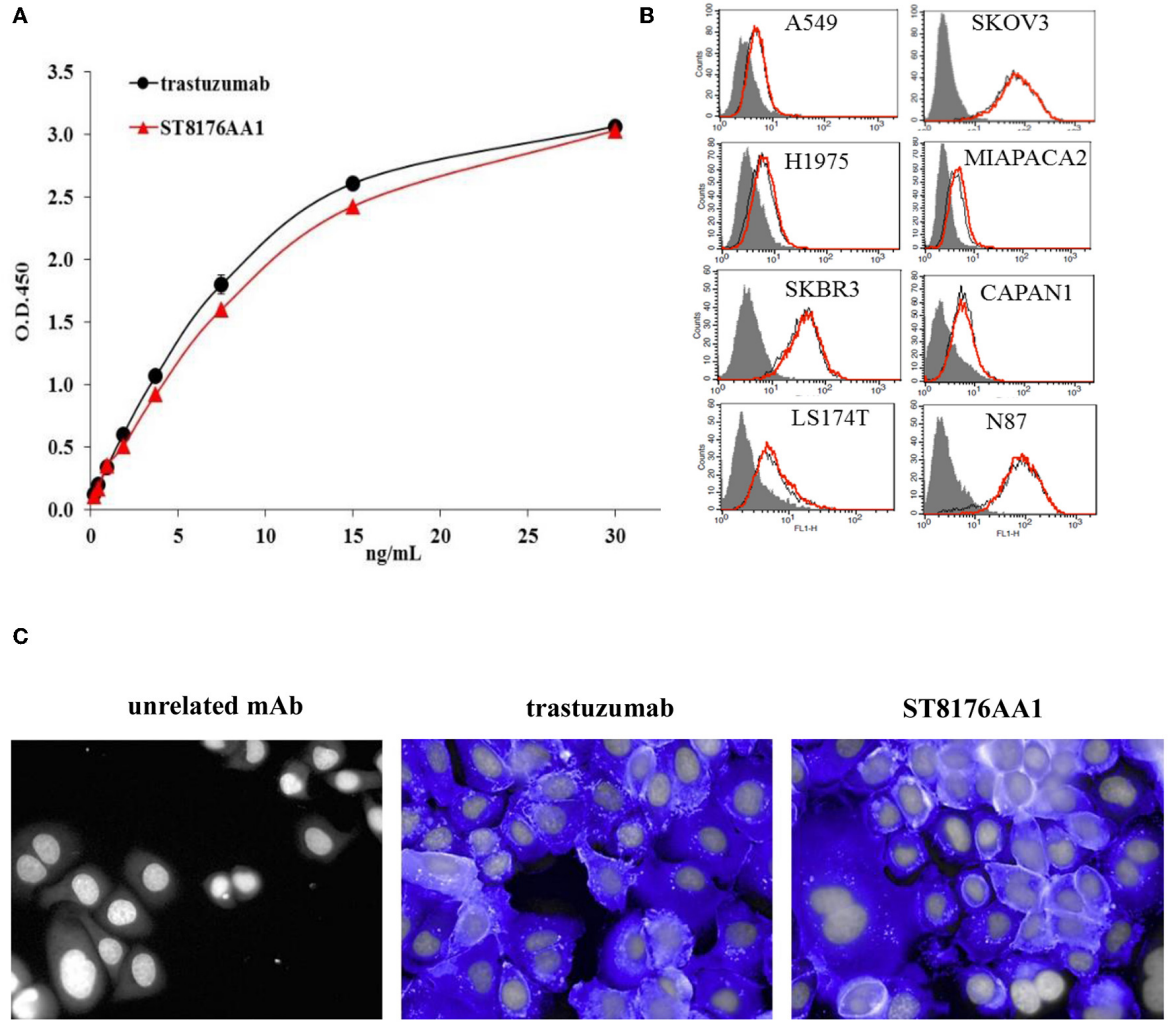
Receptor binding and cellular internalization of ST8176AA1. **(A)** Immunoreactivity of ST8176AA1 compared to trastuzumab by ErbB2-specific ELISA. Results are the mean of two independent experiments ± SD. **(B)** Binding of ST8176AA1 (red line) compared to trastuzumab (black line) by cytofluorimetry to human lung (NCI-H1975 and A549), breast (SKBR3), pancreas (Capan-1 and MiaPaCa2), ovary (SKOV3), colon (LS174T), and gastric (N87) carcinoma cell lines. Cell pellets were incubated with test items and then stained with FITC-conjugated mouse anti-human Ig and propidium iodide. Gray peaks refer to cells without primary antibody. **(C)** Internalization of ST8176AA1 and trastuzumab (both at 5 μg/mL) by SKBR3 breast cancer cells, as measured by HCS fluorescence imaging after 1 h incubation. After washing, the cells were fixed and stained by using FITC-conjugated mouse anti-human Ig (blue signal). Draq5 dye staining of nucleus (gray). Each image is representative of at least five fields of duplicate wells. Magnification 60X. Data are from one representative experiment out of two.

**Figure 3:**
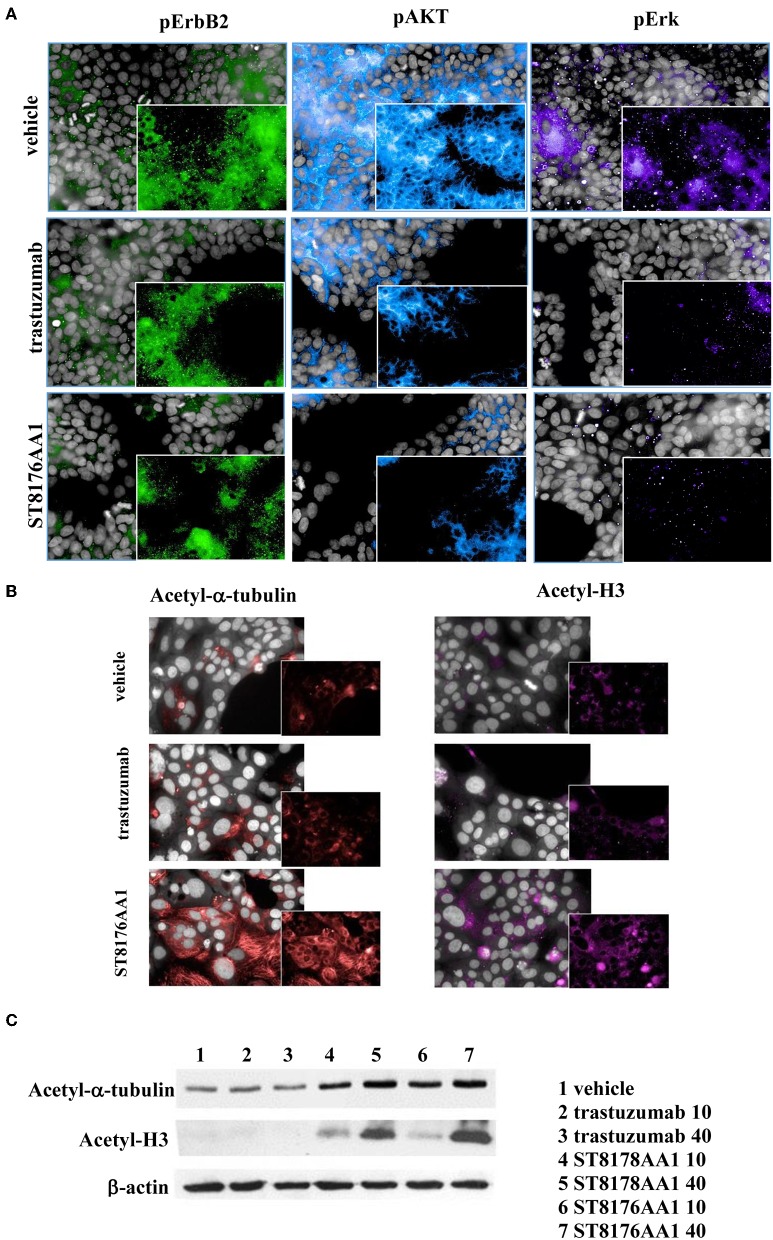
ST8176AA1 inhibits ErbB2 signaling and upregulates acetylation of histones and alpha-tubulin in tumor cells. **(A)** HCS imaging showing phosphorylation of ErbB2 and downstream molecules in heregulin (20 ng/mL)-activated LS174T human colon carcinoma cells. ST8176AA1 and trastuzumab were used at 30 μg/mL (24-h incubation). **(B)** HCS imaging showing acetylation of alpha-tubulin (red) and histone H3 (fuchsia) in Capan-1 (pancreas carcinoma) cells. ST8176AA1 and trastuzumab were used at 5 μg/mL (3 h incubation). All panels, insets show specific fluorescence signals within the cells. Draq5 dye staining of nucleus (gray). Each image is representative of at least five fields of duplicate wells. Magnification 60X. Data are from one representative experiment out of two. **(C)** Effect of trastuzumab (lanes 2–3), ST8178AA1 or ST8176AA1 (lanes 4–5 and 6–7, respectively) at 10 or 40 μg/mL on acetylation of α-tubulin and histone H3 in LS174T cells after 3 h incubation. β-actin was used for normalization. One representative blot is shown.

The immunoreactivity and binding selectivity of ST8176AA1 was tested by ELISA on recombinant ErbB2 extracellular domain coated plates and by cytofluorimetry on ErbB2 expressing tumor cell lines, respectively. ELISA results in Figure 2A show similar immunoreactivity of ST8176AA1 and trastuzumab and the result was consistent with the pattern of tumor cell binding by cytofluorimetry (Figure 2B). High Content Screening (HCS) fluorescence imaging showed that, upon binding, both trastuzumab and ST8176AA1 are rapidly internalized by ErbB2-expressing tumor cells and are both localized within the cytoplasm (Figure 2C and Supplementary Figure S6). To address the kinetics of internalization, HCS fluorescence image acquisition was performed at different time points on several cell lines showing that ADC internalization, like trastuzumab, is already visible after 5 min and intracellular localization persists at least up to 24 h (data not shown). Phosphorylation of ErbB2, as well as phosphorylation of the downstream molecules AKT (pAKT) and ErK (pERK), were similarly inhibited by trastuzumab and ST8176AA1 in heregulin-activated LS174T cells (Figure 3A). On the other hand, ST8176AA1 but not trastuzumab induced significant acetylation of histones and alpha-tubulin in ErbB2+, but not in ErbB2- (data not shown), tumor cells of different origin (Figure 3B and Supplementary Figure S7). Acetylation data by HCS Imaging Analysis were confirmed by Western blotting in LS174T cells and results in Figure 3C indicate a slightly higher activity of ST8176AA1 compared to ST8178AA1 at inducing acetylation of both α-tubulin and histones. This difference could be related to the higher ErbB2 receptor affinity of ST8176AA1 compared to ST8178AA1 (Figure 1C).

### ST8176AA1 *in vitro* Anti-tumor Biological Activity

Inhibition of ErbB2-dependent proliferation of 32D_B2/B3 cells as well as inhibition of ErbB2-expressing tumor cell growth by ST8176AA1 was higher than that obtained with trastuzumab as measured by CellTiter-Glo Luminescent Cell Viability assay (Table 1).

**Table 1:** ST8176AA1 inhibits more than trastuzumab ErbB2-dependent cell proliferation.

	**32D B2/B3**	**32D EGFR**	**SKOV3**	**HCT116**	**LS174T**	**IGROV1**	**CAOV3**
	**+++**	**–**	**+++**	**++**	**++**	**+**	+/−
Trastuzumab	36.7 ± 5.0	1.2 ± 1.0	8.1 ± 8.0	2 ± 0.7	4.1 ± 3.6	3.8 ± 1.4	9.3 ± 1.4
ST8176AA1	43.2 ± 1.5	0.2 ± 1.0	^***^66 ± 4.9	*51.6 ± 6.0	^***^59.8 ± 8.7	*38.9 ± 2.6	^***^34.7 ± 1.6

*Antiproliferative activity of 500 nM ST8176AA1 or trastuzumab, after 2-day culture of ErbB2-expressing (32D B2/B3) or EGFR-expressing (32D EGFR) mouse 32D cells, and after 6-day culture of ErbB2+ human ovary (SKOV3, IGROV1, CAOV3) or colon (HCT116, LS174T) carcinoma cell lines. Basal ErbB2 expression scored by cytofluorimetry (**–** for negative; +, ++, +++ for increasingly positive). Data, expressed as percentage of inhibition of cell proliferation with respect to vehicle-treated cells, are the mean (± SD) of at least two independent experiments*.

*** P < 0.001 and *P < 0.05 vs. trastuzumab (Mann-Whitney's test).

**Figure 4:**
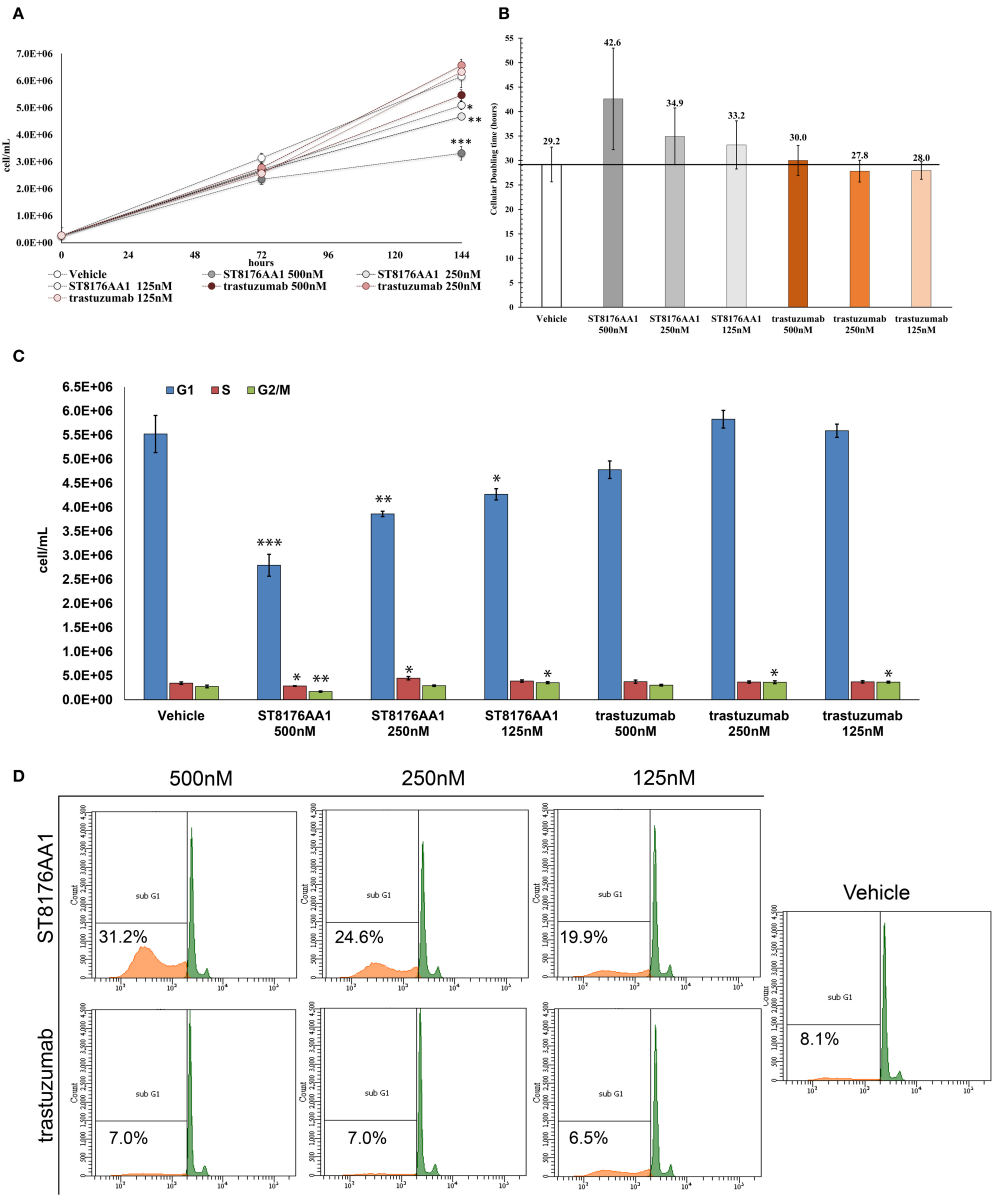
ST8176AA1 inhibits tumor cell proliferation and induces apoptosis by perturbing the cell cycle. **(A)** LS174T cells were counted by nucleocounter at indicated time points after exposure to ST8176AA1 or trastuzumab (both at 500, 250, and 125 nM). Data are the average ± SD (*n* = 3) of counted cells. **(B)** Cellular doubling time (hours) calculated in the experimental conditions as in **(A)**. **(C)** Assessment of cell cycle phases after 6 days cultivation as in **(A)** and staining with PI. Bar graph of the average ± SD (*n* = 3) of the number of cells/ml in G0/G1 (blue), S (red), and G2/M (green) phases gated on total events. **(D)** Flow cytometry of cells stained with PI after 6-day cultivation as in **(A)**. The Sub-G1 fraction of total cell population in orange. Representative results of one out of three independent samples. Student's *t*-test: ^***^P ≤ 0.001; ^**^P ≤ 0.01, and ^*^P ≤ 0.05 vs. vehicle.

**Figure 5:**
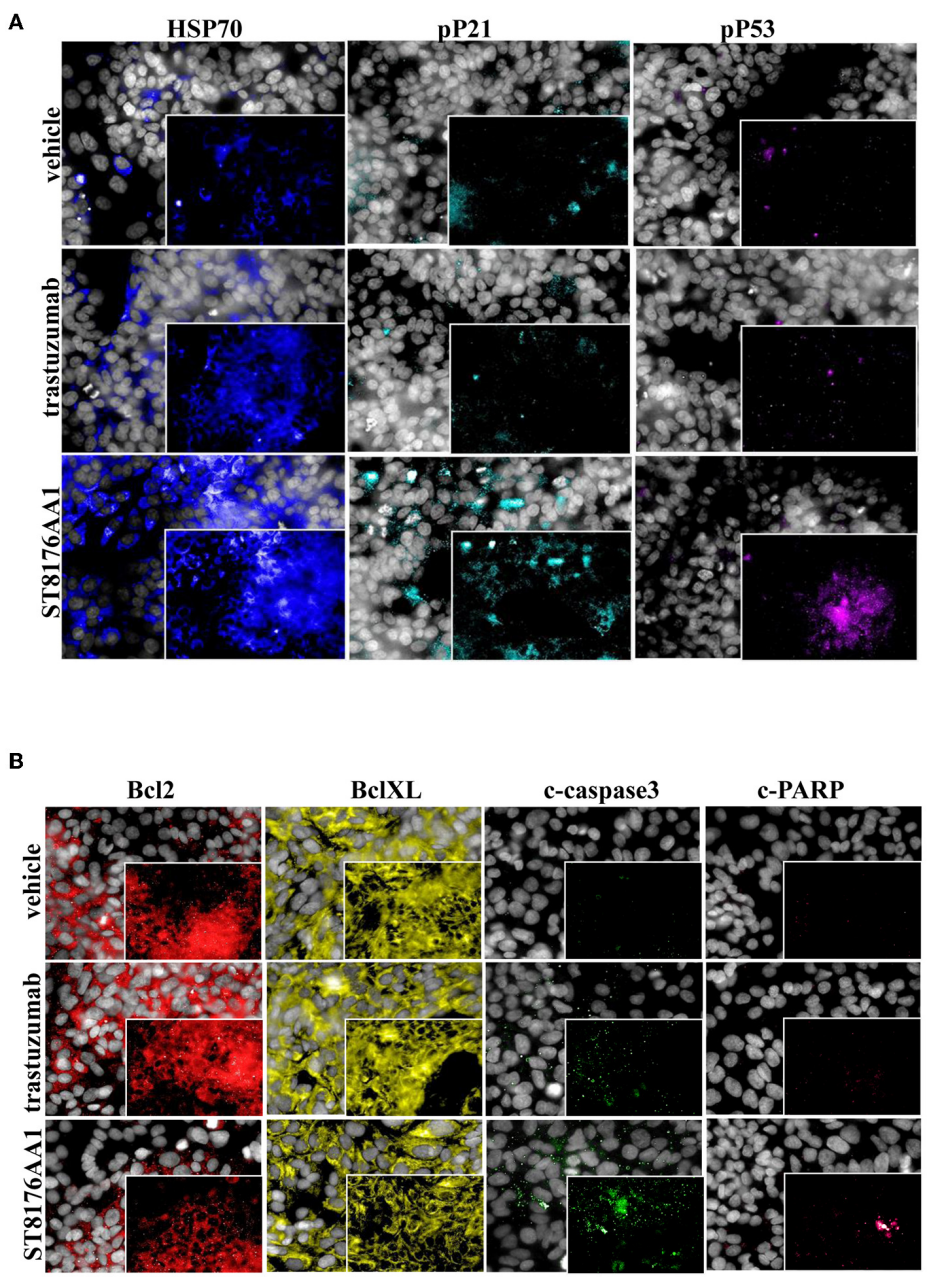
ST8176AA1 induces cell damage and reduces apoptosis resistance in tumor cells. **(A)** HCS imaging analysis showing that LS174T cells treated with ST8176AA1 exhibit increased expression of damage markers HSP70, phospho-P21 (pP21) and phospho-P53 (pP53), compared to trastuzumab. ST8176AA1, and trastuzumab were used at 5 μg/mL (3 h incubation). **(B)** HCS imaging analysis showing that SKOV3 cells treated with ST8176AA1 exhibit reduced expression of the apoptosis resistance biomarkers Bcl2 and BclXL and increased pro-apoptotic cleaved-caspase3 and cleaved-PARP proteins, compared to trastuzumab. ST8176AA1 and trastuzumab were used at 40 μg/mL (3 days incubation). In all panels, insets show specific fluorescence signals within the cells. Draq5 dye staining of nucleus (gray). Each image is representative of at least five fields of duplicate wells. Magnification 60X. Data are from one representative experiment out of two.

The proliferation of wild type 32D cells that do not express ErbB receptors, and of 32D_EGFR cells, which express EGFR but not ErbB2, was not inhibited, thus confirming the ADC selectivity for ErbB2-expressing cells. Notably, the extent of antiproliferative effect of ST8176AA1 correlated with the level of ErbB2 expression. Inhibition of LS174T cell growth by ST8176AA1 was confirmed by cell counting (Figure 4A). Coherently with these data, the cellular doubling time resulted dose-dependently increased upon treatment with ST8176AA1, but not with trastuzumab (Figure 4B). At the dose of 500 nM, ST8176AA1 induced a cellular doubling time of about 43 h while in vehicle- or trastuzumab-treated cells it was calculated to be about 30 h. Cell cycle analysis of propidium iodide (PI)-stained cells showed a significant and dose-dependent reduction of the cell fraction in G1 phase upon 6-day treatment with ST8176AA1 paired, at the maximum dose, by a strong reduction of the cell fraction in G2/M phase, while the presence of cells in S phase was differently affected at the two highest doses (Figure 4C). A similar trend was observed at 72 h (data not shown). It is noteworthy that measurements performed at two fixed time points (72 and 144 h) only allow to observe that the completion of cell cycle seems impaired in ST8176AA1-treated cells and that the cells likely become programmed to death, but observations are not sufficient to establish which phase of the cell cycle is really impaired and when it occurs, as well as whether the observed phenomenon is associated to a cytotoxic rather than cytostatic effect. Anyhow, consistently with the cell cycle perturbations described above, the percentage of apoptotic cells (PI-stained) was significantly and dose-dependently higher when the cells were cultivated in the presence of ST8176AA1 compared to trastuzumab or vehicle (Figure 4D). Furthermore, the perturbation of LS174T cell cycle by ST8176AA1 was associated to the induction of DNA damage biomarkers, such as phosphorylated P53 and P21, as well as HSP70 (Figure 5A). The pro-apoptotic effect of ST8176AA1 was also confirmed in SKOV3 cells, which exhibited lower expression of the anti-apoptotic effector proteins Bcl-2 and Bcl-XL and increased expression of cleaved-caspase-3 and cleaved-PARP proteins when treated with the ADC compared to trastuzumab (Figure 5B).

**Figure 6:**
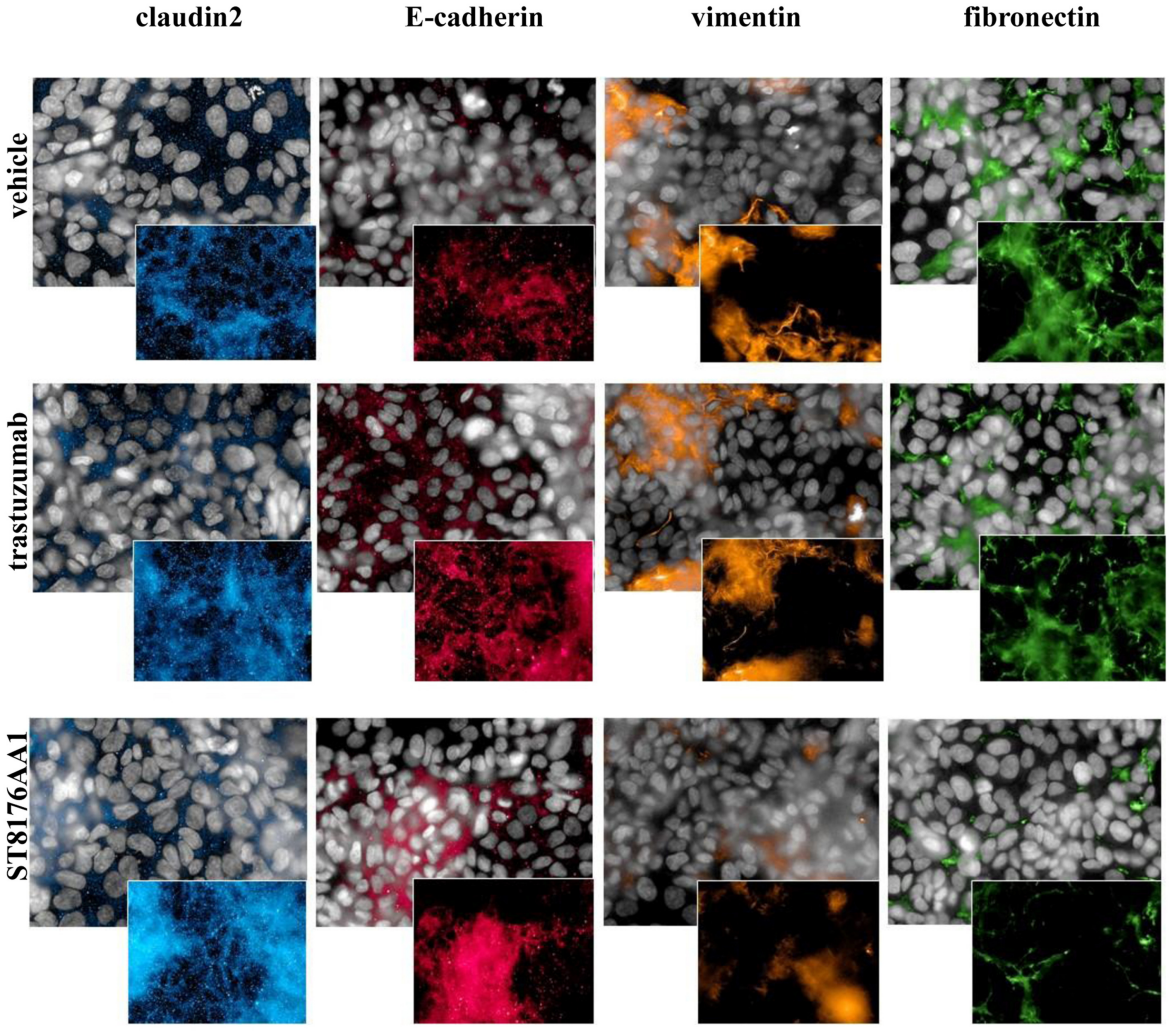
ST8176AA1 induces reversal of epithelial/mesenchymal transition in tumor cells. HCS imaging analysis showing that SKOV3 cells treated with ST8176AA1 exhibit increased expression of the claudin2 and E-cadherin (epithelial) proteins and reduction of vimentin and fibronectin (mesenchymal) proteins. ST8176AA1 and trastuzumab were used at 15 μg/mL (3 days incubation). In all panels, insets show specific fluorescence signals within the cells. Draq5 dye staining of nucleus (gray). Each image is representative of at least five fields of duplicate wells. Magnification 60X. Data are from one representative experiment out of two.

Epithelial Mesenchimal Transition (EMT) is a key step into tumor invasion and metastatization that has been previously shown to be counteracted by HDACi ST7612AA1 (13). Therefore, we assessed the effects of ST8176AA1 on typical epithelial/mesenchymal markers in SKOV3 and LS174T cells by HCS Imaging analysis. As shown in Figure 6 (SKOV3) and in Supplementary Figure S8 (LS174T), up-regulation of the epithelial markers claudin-2 and E-cadherin and down-modulation of the mesenchymal markers vimentin and fibronectin were clearly achieved when cells were incubated with ST8176AA1, but little if any effect was obtained with trastuzumab, thus confirming the ability of ST8176AA1 to counteract EMT of tumor cells clearly dependent on epigenetic modulation through the release of the active HDAC inhibitor.

**Figure 7:**
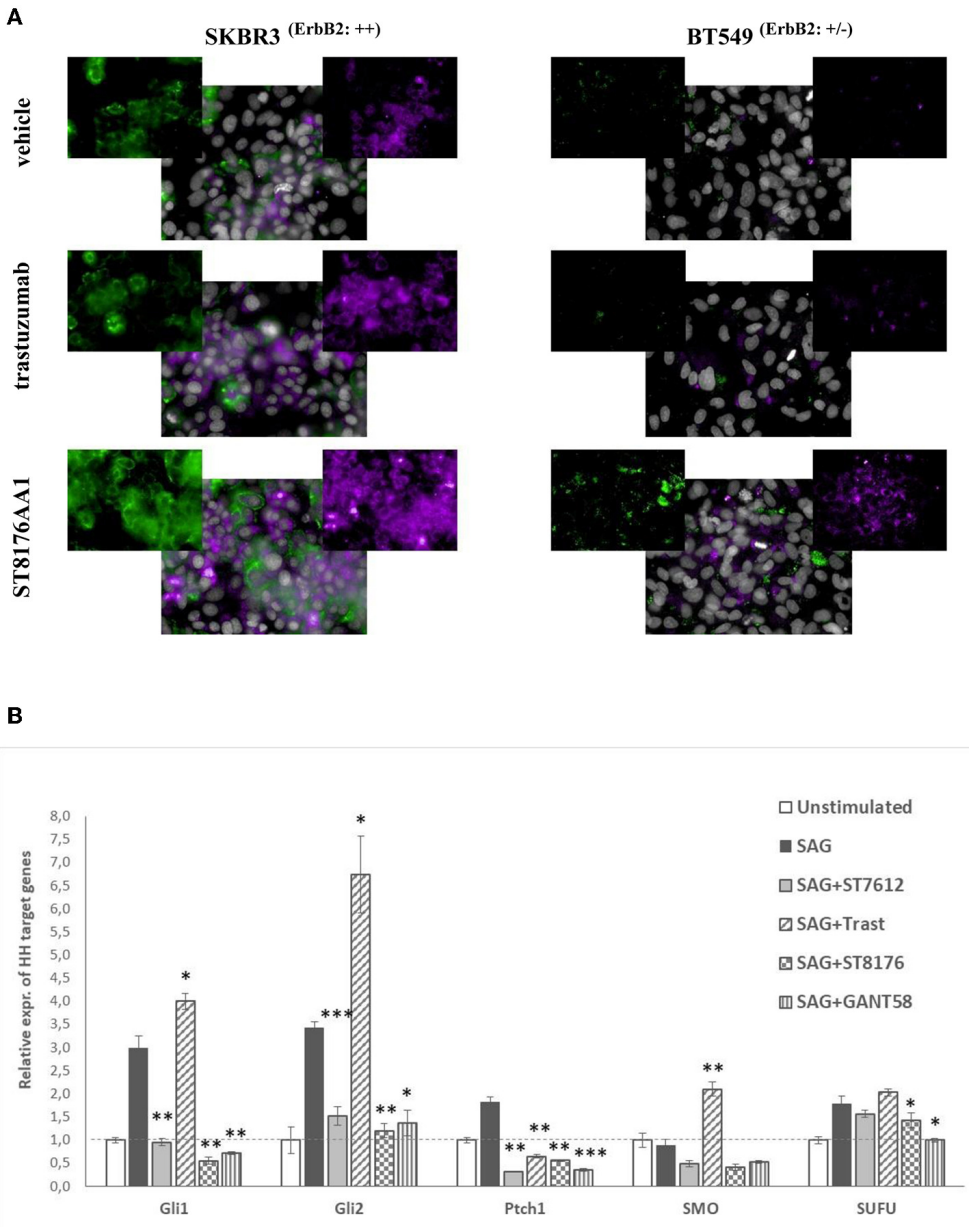
ST8176AA1 induces the expression of ERα and ErbB2 in ErbB2+ and triple negative breast cancer cells. **(A)** HCS fluorescence imaging analysis show that ErbB2-positive (SKBR3) and triple negative (BT549) breast cancer cells treated with ST8176AA1 exhibit increased expression of ERα (purple) and ErbB2 (green) proteins, compared to trastuzumab. ST8176AA1 and trastuzumab were used at 5 μg/mL (3 days incubation). Basal ErbB2 expression scored by cytofluorimetry in brackets. In all panels, insets show specific fluorescence signals within the cells. Draq5 dye staining of nucleus (gray). Each image is representative of at least five fields of duplicate wells. Magnification 60X. Data are from one representative experiment out of two. **(B)** SAG-induced Hh target gene expression measured by qPCR in LS174T cells after 24 h-treatment with ST7612AA1 (100 nM), ST8176AA1 or trastuzumab (250 nM) and with the reference Hh inhibitor GANT58 (10 μM). Graph displays the relative expression of the indicated target genes (after normalization to the housekeeping gene GUSB) as compared to not stimulated cells. Data are mean of triplicates ± SD. Student's *t*-test: ^***^P ≤ 0.001; ^**^P ≤ 0.01, and ^*^P ≤ 0.05 vs. SAG.

Epigenetic modulators have been previously shown to enhance the response of triple negative breast cancer (TNBC) cells to hormonal therapy through restoration or upregulation of the Estrogen Receptor-α (ERα) (19, 20). Accordingly, we wondered whether ST8176AA1-mediated epigenetic modulation could also induce up-regulation of ERα expression in breast cancer cell lines. HCS fluorescence imaging clearly showed increased ErbB2 and ERα expression by ST8176AA1 but not trastuzumab in SKBR3 and MCF7 cells which are ErbB2 and ER positive but also, and more importantly, induced expression of both receptors in triple negative BT549 and MDA-MB231 cells (Figure 7A and Supplementary Figure S9, respectively). Basal ErbB2 expression in each cell line was scored by flow cytometry with trastuzumab and representative histograms are shown in Supplementary Figure S10.

In the last decade, several papers have highlighted the role of HDAC1/2 as regulators of the Hedgehog (Hh) signaling in tumor cells, through deacetylation and transcriptional activation of Gli1 and Gli2 proteins and, consequently, the possible use of HDACis as potential modulators of this pathway in some types of tumors (21, 22). Starting from these observations, we investigated the putative effect of ST8176AA1-mediated epigenetic modulation on the Sonic hedgehog (Hh) signaling pathway and, in particular, on Gli1 transcriptional activity, in colon carcinoma cells where activation of this pathway plays a relevant role. LS174T cells, activated with the known Hh agonist SAG (Smoothened agonist) (23), were cultivated for 24 h with 250 nM ST8176AA1 or trastuzumab, as well as with the reference HDACi ST7612AA1 (100 nM) or the known Hh inhibitor GANT58 (10 μM), and then the relative expression of Hh target genes was measured by real time qPCR. Data in Figure 7B show that SAG-induced upregulation of the Hh pathway genes is efficiently counteracted by the Hh-inhibitor GANT58, as expected. Surprisingly, trastuzumab induced a significant increase of transcription of 3 (Gli1, Gli2, and SMO) out of 5 SAG-induced Hh target genes, whereas for the other two genes a decrease (Ptch1) or no effect (SUFU) was measured. On the contrary, similarly to ST7612AA1, ST8176AA1 clearly counteracted the SAG-induced upregulation of gene transcription likely as a consequence of epigenetic modulation mediated by the active HDACi.

### ST8176AA1 Exhibits Anti-tumor Activity in *in vivo* Tumor Models

**Figure 8:**
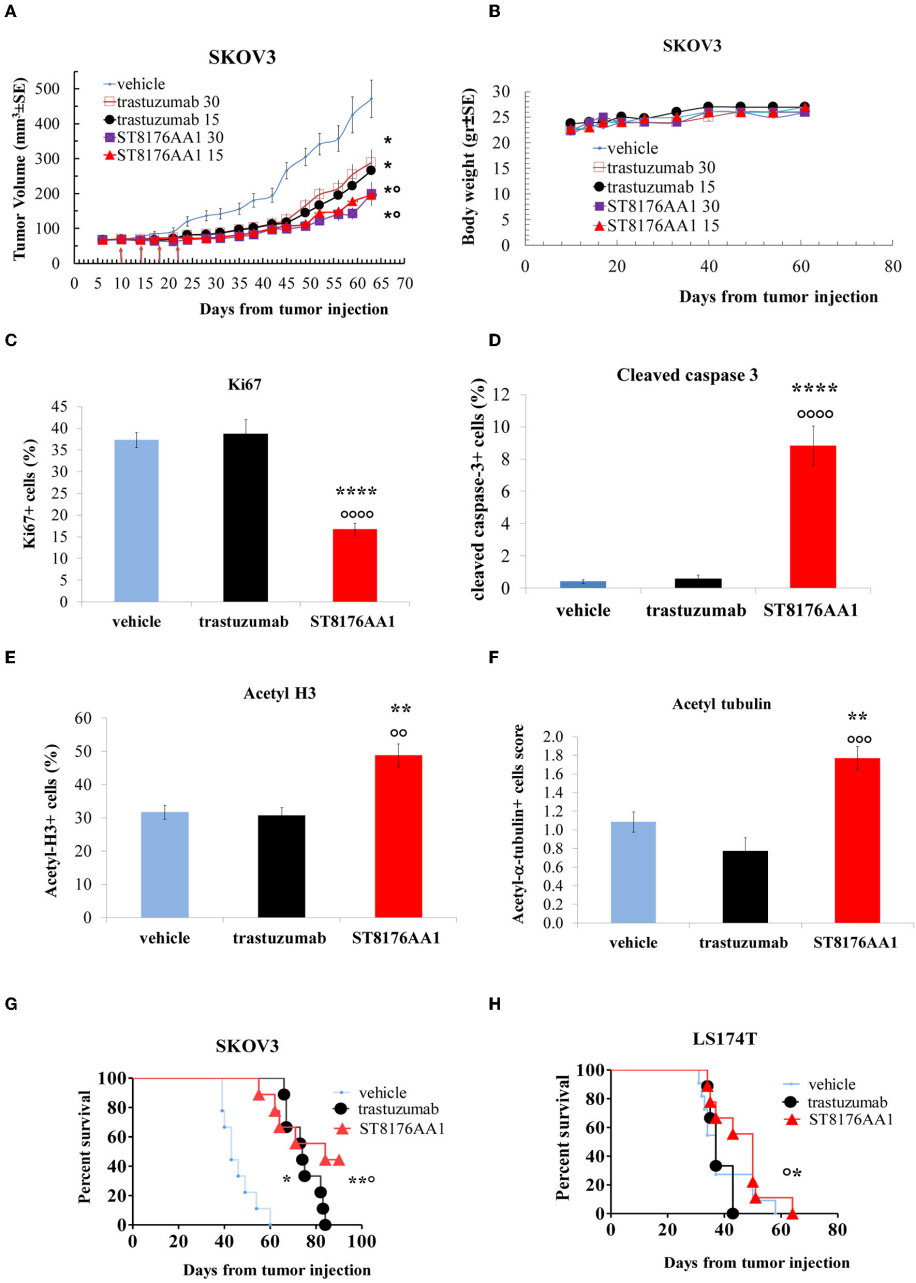
Anti-tumor efficacy of ST8176AA1 in tumor xenografts. **(A)** Subcutaneous tumors were induced in nude mice (10/group) by injecting human SKOV3 cells ovary carcinoma. When tumor masses reached an average size of 50 mm^3^ mice were treated i.p. (4 doses of 15 or 30 mg/kg once every 4 days, starting 10 days after tumor cell transplantation) with ST8176AA1 or trastuzumab. One group received vehicle (PBS) with the same schedule. Tumor growth was monitored using a Vernier caliper. **(B)** Body weight. Data are the mean ± SE. Statistical analysis by Mann-Whitney's test *P < 0.05 vs. vehicle and °*P* < 0.05 vs. trastuzumab; **(C–F)** Tumor masses at the end of study in **(A)** were analyzed by immunohistochemistry. Cells positive for Ki67 **(C)**, cleaved-caspase 3 **(D)**, acetyl-H3 **(E)**, acetyl-α-tubulin **(F)** were counted by two independent observers in five randomly selected fields. Data in the graph are expressed as the mean of positive cells × 100/total cells ± SE or as score with negative staining, score 0; 1-20% positive cells, score 1+; 21-50% positive cells, score 2+ >50% positive cells, score 3+. Statistical analysis by Mann-Whitney's test. **P < 0.01 and ****P < 0.0001 vs. vehicle-treated group; °°*P* < 0.01, °°°*P* < 0.001, and °°°°*P* < 0.0001 vs. trastuzumab. **(G)** Orthotopic tumor models of ovarian and colon **(H)** cancer by tumor cell injection in the peritoneum of mice (10/group). Mice were treated ip with ST8176AA1 or trastuzumab (4 doses of 15 mg/kg once every 4 days, starting 3 days after tumor cell transplantation). One group received vehicle (PBS) with the same schedule. Median survival time and Kaplan Myers were evaluated. Data are expressed as mean ± SE, *P < 0.05 and **P < 0.01 vs. vehicle and °*P* < 0.05 vs. trastuzumab.

Based on *in vitro* results showing that ST8176AA1 was able to induce tumor cell damage and apoptosis by exerting epigenetic modulation, we investigated *in vivo* anti-tumor effects of the compound in different tumor models. In the subcutaneous ErbB2+ ovarian carcinoma SKOV3 xenograft model, ST8176AA1 at 15 or 30 mg/kg doses administered intraperitoneally (ip) four times every 4 days (q4dx4), starting 10 days after tumor transplantation, induced a statistically significant higher tumor growth inhibition compared to vehicle or trastuzumab (Figure 8A). No variation of the animal body weight indicated good tolerability of the treatment (Figure 8B). Immunohistochemistry evaluations of excised tumors were carried out 24 h after one last dose of ST8176AA1 (15 mg/Kg group). Scoring of positive staining was performed in randomly selected fields of the tissue slides by two independent observers who were blinded to the treatments. In agreement with tumor growth inhibition, immunohistochemistry showed significant reduction of Ki67+ cells (Figure 8C) and a higher number of cleaved-caspase 3+ (Figure 8D), as well as acetyl-H3+ (Figure 8E) and acetyl-α tubulin+ (Figure 8F) cells, in the tumors from mice treated with ST8176AA1 compared to either vehicle or trastuzumab. Interestingly, the trastuzumab anti-tumor activity was not associated to variation of Ki67+, cleaved-caspase 3+, acetyl-H3+ or acetyl-α-tubulin+ cells thus confirming the different mechanisms of action of the antibody compared to ST8176AA1.

In a subsequent study, SKOV3 cells were transplanted intraperitoneally to simulate a possible human condition of ovary peritoneal carcinoma. ST8176AA1 and trastuzumab were administered intraperitoneally at the dose of 15 mg/kg, according to the schedule q4dx4. Results in Figure 8G show that ST8176AA1 induced a median survival time (MST) of 84 days compared to 43 and 74 days of vehicle- and trastuzumab-treated groups, respectively. These results are consistent with the previous study confirming that ST8176AA1 is statistically significant more effective than trastuzumab against ovary carcinoma.

**Figure 9:**
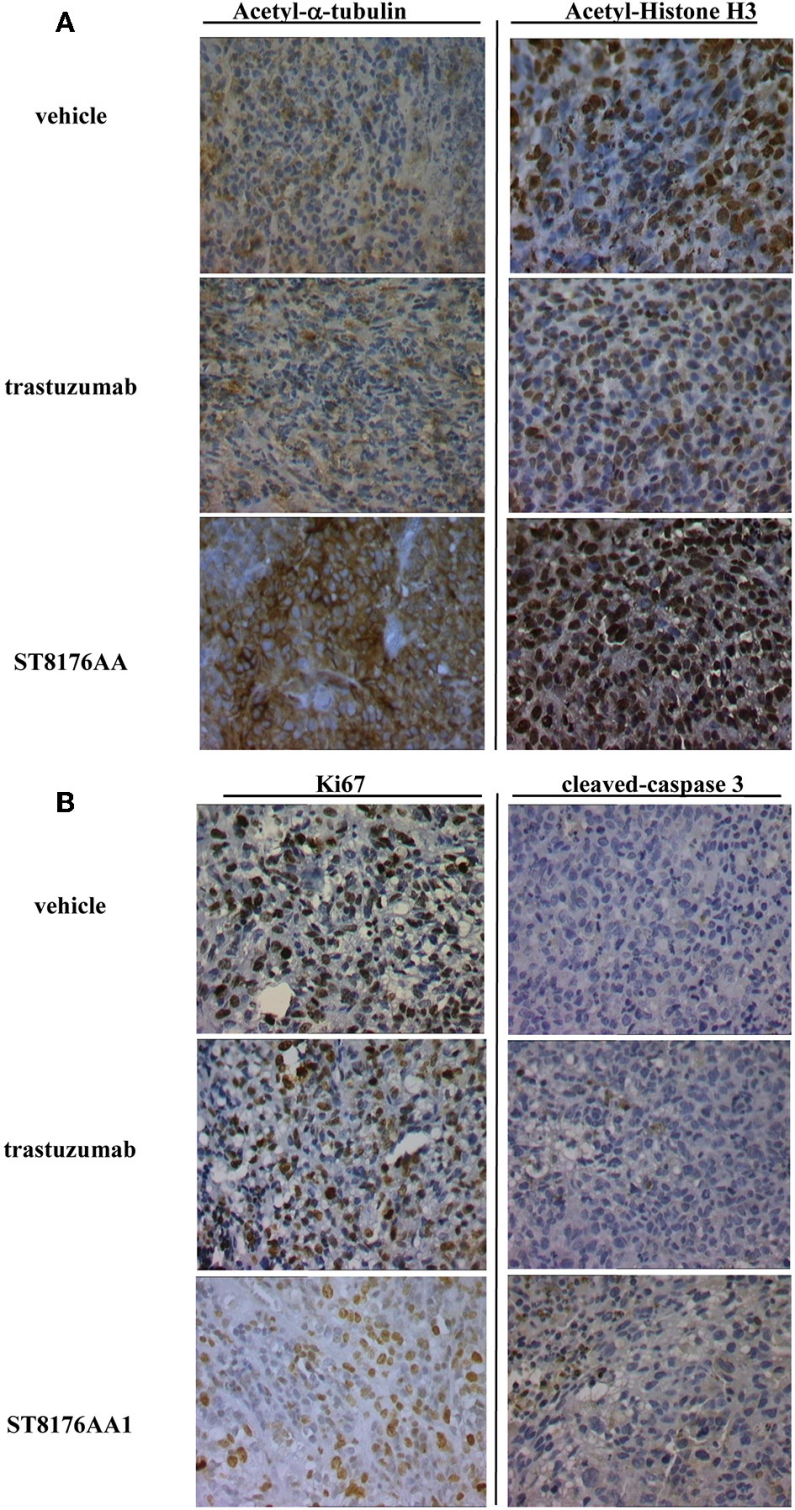
Immunohistochemistry of tumor masses from SKOV3 xenografts. SKOV3 xenografts collected 24 h after the last drug administration and analyzed by immunohistochemistry for the expression of acetylated-α-tubulin and acetylated-histone H3 **(A)**, or Ki67 and cleaved-caspase-3 **(B)**. Images captured using 40 × magnification. Scale bar: 500 μm. One representative section, out of 6, for each group is shown.

Anti-tumor efficacy was further investigated in a model of LS174T colon carcinoma implanted intraperitoneally. In this case, ST8176AA1 but not trastuzumab induced statistically significant improvement of survival (MST = 50 days vs. vehicle- or trastuzumab-treated groups MST = 37 days) (Figure 8H). Pictures of immunohistochemistry staining of one representative section from each group of the study of Figure 8A are given in Figures 9A,B.

**Figure 10:**
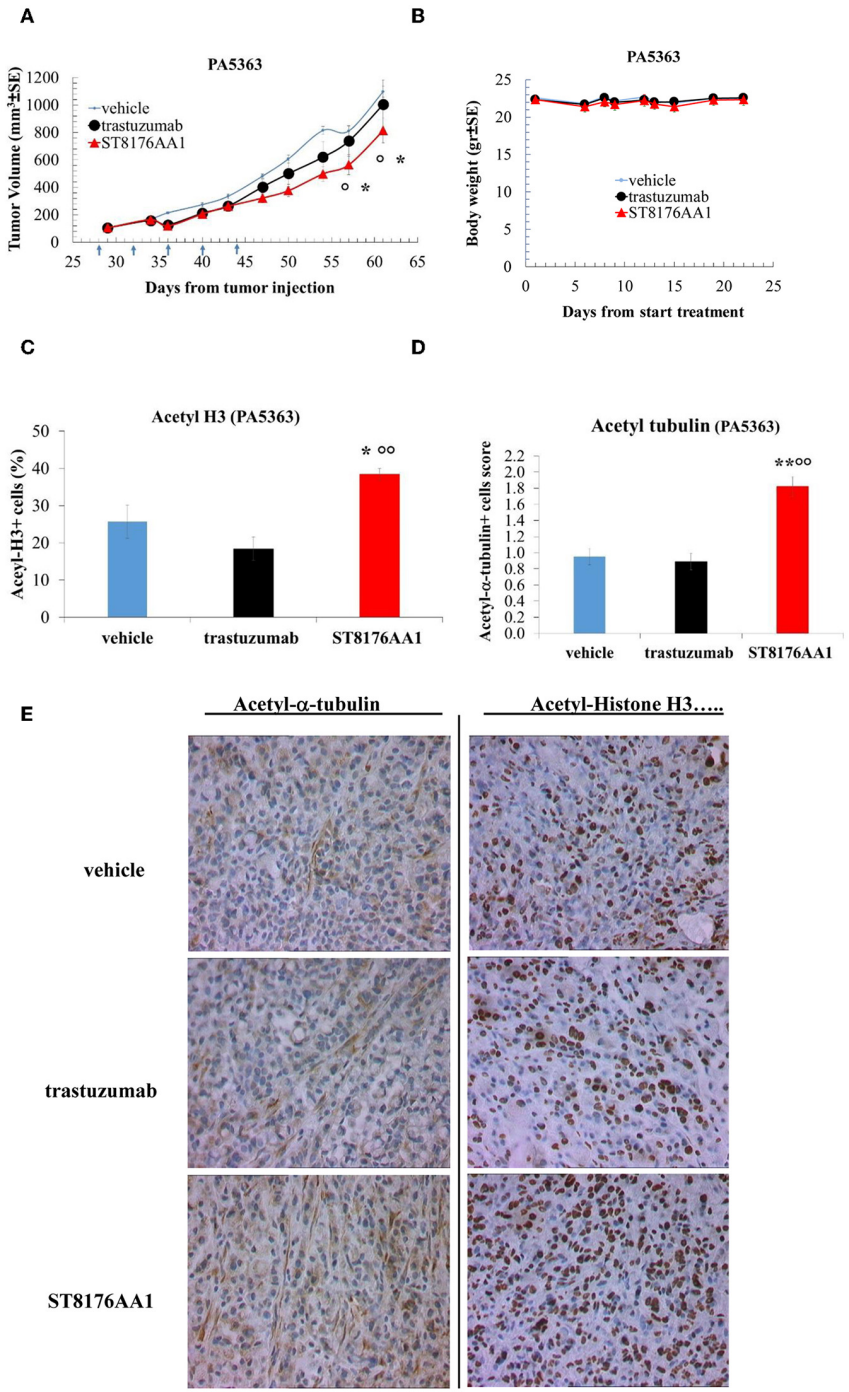
Anti-tumor efficacy of ST8176AA1 in PDX pancreatic carcinoma model of patient PA5363. **(A)** NODSCID mice (10/group) bearing patient-derived pancreatic carcinomas PA5363 were treated i.p. with ST8176AA1 or trastuzumab (5 doses of 15 mg/kg once every 4 days, starting 28 days after tumor transplantation as indicated by arrows in the graph). One group received vehicle (PBS) with the same schedule. Tumor growth was monitored using a Vernier caliper. Body weight in panel **(B)**. Data are expressed as mean ± SE. Statistical analysis by Mann-Whitney's test. *P < 0.05 vs. vehicle and °*P* < 0.05 vs. trastuzumab. **(C,D)** Tumor masses at the end of the study were analyzed by immunohistochemistry. Cells positive for acetyl-histone H3 **(C)** and for acetyl-α-tubulin **(D)** from PA5363 were counted by two independent observers in five randomly selected fields. Data in the graph are expressed as the mean of positive cells × 100/total cells ± SE or as score with negative staining, score 0; 1-20% positive cells, score 1+; 21-50% positive cells, score 2+ >50% positive cells, score 3+. Statistical analysis by Mann-Whitney's test. *P < 0.05 vs. vehicle-treated group; °°*P* < 0.01 vs. trastuzumab. **(E)** Representative immunohistochemistry pictures for acetyl-α-tubulin and acetyl-histone H3 are shown. Images captured using 40 × magnification. Scale bar: 500 μm.

**Figure 11:**
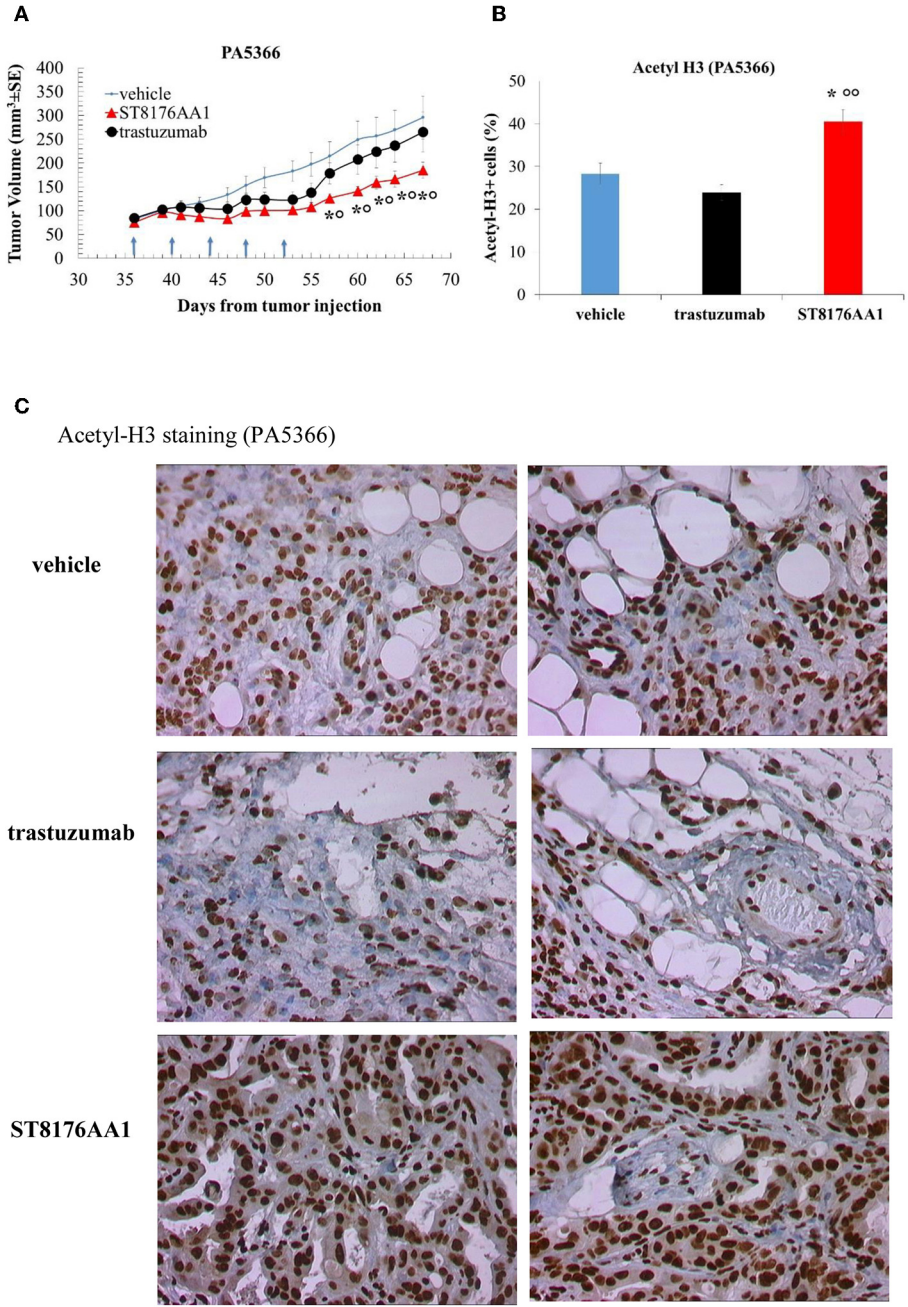
Anti-tumor efficacy of ST8176AA1 in PDX pancreatic carcinoma model of PA5366. **(A)** NODSCID mice (10/group) bearing patient-derived pancreatic carcinomas PA5366 were treated i.p. with ST8176AA1 or trastuzumab (5 doses of 15 mg/kg once every 4 days, starting 36 days after tumor transplantation as indicated by arrows in the graph). One group received vehicle (PBS) with the same schedule. Tumor growth was monitored using a Vernier caliper. Data are expressed as mean ± SE. Statistical analysis by Mann-Whitney's test. *P < 0.05 vs. vehicle and °*P* < 0.05 vs. trastuzumab. **(B)** Tumor masses at the end of the study were analyzed by immunohistochemistry. Cells positive for acetyl-histone H3 from PA5366 were counted by two independent observers in five randomly selected fields. Data in the graph are expressed as the mean of positive cells × 100/total cells ± SE or as score with negative staining, score 0; 1-20% positive cells, score 1+; 21-50% positive cells, score 2+ >50% positive cells, score 3+. Statistical analysis by Mann-Whitney's test. *P < 0.05 vs. vehicle-treated group; °°*P* < 0.01 vs. trastuzumab. **(C)** Representative immunohistochemistry pictures for acetyl-histone H3 are shown. Images captured using 40 × magnification. Scale bar: 500 μm.

Moreover, since trastuzumab is currently being employed in clinical protocols for the treatment of pancreatic ductal adenocarcinoma in combination with chemotherapy (24) or with an HDACi (25), the anti-tumor activity of ST8176AA1 was investigated in two patient-derived xenograft (PDX) pancreatic carcinoma models. Both tumors had been previously ranked as HER2 overexpressing by gene expression analysis (RNAseq1 HER2 was 6,355 and 6,589 log2 FPKM, respectively). Results in Figures 10A,B indicate statistically significant higher anti-tumor activity of ST8176AA1 compared to trastuzumab or vehicle without any body weight loss. IHC confirmed epigenetic modulation as measured by increased H3 and α-tubulin acetylation in PDX of PA5363 (Figures 10C,D). Pictures of immunohistochemistry staining of one representative section of each biomarker are given in Figure 10E. Results were confirmed in the second PDX PA5366 (Figure 11A) correlating with increased acetylation of H3 (Figures 11B,C). Acetylation of α-tubulin was not evaluated for insufficient tumor material.

## Discussion

Upon approval of Adcetris in 2011, Kadcyla in 2013, Besponsa and Mylotarg (re-approval) in 2017, the recombinant immunotoxin Lumoxiti in 2018 and Polivy in 2019, the antibody drug conjugate field is seeing a tremendous increase in the number of drug candidates in pre-clinical and clinical development with more than 80 ADCs in clinical trials for different indications (26, 27). As of today, of the five marketed products, only Kadcyla is targeting solid tumors. Kadcyla is made of trastuzumab conjugated via a non-cleavable linker to the microtubule disrupting agent emtansine and it is approved for the treatment of metastatic ErbB2-expressing breast and gastric cancer. There are several factors affecting efficacy and tolerability of ADCs including antibody target relevance and binding properties, cellular uptake, release of the active moiety, and relevance of the targeted molecular pathway in the maintenance of tumor malignancy (28). The role of the antibody appears to be particularly important, in fact ADCs made of two different antibodies targeting the same antigen (i.e., CD79b) and conjugated to the same toxin can exhibit different safety and efficacy profiles in patients (26). Therefore, to investigate new ADCs, we decided to exploit well-known monoclonal antibodies like cetuximab and trastuzumab which are clinically validated having been used for decades for the treatment of solid tumors. We recently described ADCs made of cetuximab conjugated to a payload derived from the HDACi ST7612AA1 and exhibiting epigenetic modulation that induced anti-tumor activity (11). Here we describe for the first time the ADC ST8176AA1 made of trastuzumab conjugated via a non-cleavable linker to the same HDACi moiety. Data show that ST8176AA1 substantially maintains the antigen recognition properties of trastuzumab both in terms of immunoreactivity and affinity. Superior anti-tumor activity of ST8176AA1, delivered parenterally to mice bearing tumor xenografts of human ovarian, colon or pancreas carcinoma has been demonstrated in comparison with trastuzumab. The *in vivo* anti-tumor activity of ST8176AA1 correlated with the induction of epigenetic modulation in ErbB2+ tumor cells as measured both *in vitro* and *in vivo* by increased acetylation of histones and tubulin. Ovarian cancer is one of the most frequent cause of cancer-related deaths and chemotherapy remains the main option for its management (Cancer Genome Atlas Research Network). Despite frequent preliminary responses to chemotherapy, this tumor often relapses, and limited alternative agents are currently available. Recently, investigational therapeutic strategies with biological agents such as antibody-drug conjugates have been added to chemotherapy (29). In a recent clinical trial, mirvetuximab soravtansine targeting folate receptor alfa showed promising results in platinum-resistant ovarian cancer (30). Clinical efficacy in the treatment of recurrent ovarian cancer was also achieved with the combination of trastuzumab and abraxane in comparison with single drugs in terms of minor neutropenia, leukopenia and thrombocytopenia (31). To treat ovarian cancer, epigenetic therapies have been recently proposed to counteract genetic abnormalities which are particularly frequent in this type of tumor (32). Unfortunately, clinical trials with either a single epigenetic modulator or in combination with other drugs, have been disappointing for the appearance of drug-related toxicity inducing fatigue, vomiting and neutropenia (32, 33). Interestingly, the ADC ST8176AA1 here described appears to inhibit tumor growth without inducing toxicity and it could therefore be an attractive opportunity for patients with ErbB2+ ovarian cancer. In fact, treatment with ST8176AA1 induced neither body weight loss nor clinical signs or adverse events and at necropsy, no macroscopic alterations of organs were found. Moreover, in a recent paper, for the first time, HDAC2 was highlighted as a key factor regulating *in vivo* cancer growth and cancer stem cells (CSCs) phenotype involved in cancer initiation, progression and chemoresistance of osteosarcoma (34). This observation is of particular interest because the HDACi moiety of ST8176AA1 is known to be also effective against the HDAC2 isoform (13).

The development of novel targeted therapies is also particularly needed for recurrent colon and pancreatic cancer. Anti-epidermal growth factor receptor (EGFR) monoclonal antibodies such as panitumumab and cetuximab as single agents showed an activity in Kras wild-type colorectal cancer but presented limited benefits (1–2 months) (35, 36). Interestingly, ErbB2 activation is present in about 5% of colorectal cancers developing resistance to EGFR-targeted therapies thus supporting investigation of anti-ErbB2 antibodies and related ADCs in these patients (37).

We previously reported increased anti-tumor activity of ADCs ST8154AA4 and ST8155AA1 (cetuximab-HDACi) compared to cetuximab in EGFR+ tumor models including pancreatic carcinoma CAPAN-1 (11). Presently, we show the advantage of delivering HDACi to both ErbB2+ colon and pancreatic carcinomas by the ADC ST8176AA1. Present results with PDX models are particularly relevant because in these models the transplanted tumor is expected to maintain the original histology, molecular and biological properties of the patient's tumor (38, 39) and consequently, drug responses obtained from PDX mice are generally recognized as predictive of a therapeutic response in human patients (40, 41). In fact, clinical trials in which patients and related PDX mice were treated with the same drugs well-demonstrated to have parallel clinical responses (42). It is to note that ST8176AA1 appears to be an improved version of both trastuzumab and ST7612AA1 because it is shown to be effective in cases where trastuzumab is poorly if any active, by delivering an HDACi dose that is estimated to be several orders of magnitude lower than effective doses of the untargeted drug (13). Clinical translation of this ADC might be facilitated because (a) extensive clinical knowledge of trastuzumab and (b) expected much higher tolerability compared to currently approved ADCs because of safety of the effector moiety.

Therefore, present results obtained with ST8176AA1 in PDX pancreas carcinoma are of particular relevance. EGFR family is an attractive target for pancreatic carcinoma since 40% of tumors overexpress EGFR and 20% ErbB2 (43). Moreover, the overexpression of these receptors was associated with worse outcomes and a therapeutic synergism between anti-ErbB2 therapy with anti-EGFR agents was also seen in pancreatic xenografts expressing moderate or low ErbB2 (44–46). Combination therapies could be highly efficacious but gastrointestinal toxicity is a key limiting factor as reported in studies with trametinib or erlotinib (47). Genetic heterogeneity and plasticity of pancreatic tumor cells make unlikely to obtain significant and durable responses by addressing a single pathway (48), therefore the use of ST8176AA1 alone or combined with the cetuximab-HDACi could be an option worth testing. Present data also indicate for ST8176AA1 a possible use for the treatment of triple negative breast cancer. In this clinical indication, neither trastuzumab nor T-DM1 are an option because of lack or insufficient expression of ErbB2. Our results clearly demonstrate that, even in the presence of very low ErbB2 expression, ST8176AA1-mediated epigenetic modulation leads to significant up-regulation of ERα, consistently with previous results obtained with HDACi (20). Targeted epigenetic modulation could be an attractive approach to interfere with tumor malignancy and escape strategies. The possible use of HDACi has been recently postulated to up-regulate the expression of MHC class-I in protocols with immune check point inhibitors (49). Moreover, epigenetic modulators could act as activators of potential multi-targeted drugs as recently postulated for the Sonic hedgehog signaling (50) and supported by our data (Figure 7). Overall, present results indicate that ST8176AA1 is a good drug candidate to deliver epigenetic modulation to tumors via the ErbB2 receptor. ST8176AA1 could be an alternative or a companion drug to T-DM1 for the treatment of ErbB2+ tumors and a unique therapeutic opportunity for triple negative breast cancer that might be inhibited by ST8176AA1 and also be made sensitive to tamoxifen standard therapies. Overall, ST8176AA1 compared to T-DM1 appears to exhibit two major advantages: (a) it is in principle applicable for the treatment of triple negative breast cancer; (b) it lacks the intrinsic toxicity of the T-DM1 payload which has been recently reported to be a binder of the cytoskeleton-associated protein 5 on human hepatocytes, a feature that explains off-target toxicity of the ADC in patients (6).

## Conclusion

We show that ErbB2+ tumors that include but are not limited to ovary, colon or pancreas could benefit from treatments with the ADC ST8176AA1 that, by inducing epigenetic modulation through trastuzumab-mediated delivery of an HDACi, can inhibit tumor cell growth. Interestingly, ST8176AA1 also induced ErbB2 and ERα expression in triple negative breast cancer cells thus possibly making this orphan tumor, sensitive to standard treatments. ST8176AA1 is expected to have an excellent tolerability lacking the intrinsic toxicity of the trastuzumab-emtansine (T-DM1) payload and acting at HDACi dose order of magnitude lower than those generally used systemically.

## Data Availability Statement

All datasets generated for this study are included in the article/Supplementary Material.

## Ethics Statement

The animal study was reviewed and approved by OPBA Alfasigma SpA.

## Author Contributions

FM, LV, AA, CC, AR, VC, MT, EC, VF, EP, GB, LS, and GG participated in the design of experiments and execution, data acquisition, and analysis. FM, LV, GG, and RD revised data and wrote the manuscript.

### Conflict of Interest

FM, LV, AA, CC, AR, GG, GB, and RD were employed by the company Alfasigma SpA; VF was employed by the company Lead Discovery Siena srl; LS was employed by the company Fondazione Toscana Life Sciences, Siena; VC was employed by the company Histo-Cyto Service srl. The remaining authors declare that the research was conducted in the absence of any commercial or financial relationships that could be construed as a potential conflict of interest.

## Abbreviations

ADC: antibody drug conjugate
ATCC: American Type Culture Collection
AVMA: American Veterinary Medical Association
BSA: bovine serum albumin
CO_2_: carboxylic acid
CSC: cancer stem cells
DAR: drug antibody ratio
ECL: enhanced chemiluminescence
EMT: epithelial-mesenchimal transition
DHB: 2,5-Dihydroxybenzoic acid
DMEM: Dulbecco's Modified Eagle *Medium*
DMSO: dimethyl sulfoxide
DSMZ: German Collection of Microorganisms and Cell Cultures
EGFR: epidermal growth factor receptor
ELISA: enzyme-linked immunosorbent *assay*
EMEM: Eagle's Minimum Essential Medium
ErbB2: epidermal growth factor receptor 2
ErK: extracellular signal–regulated kinases
FBS: Fetal Bovine serum
FDA: Food and Drug Administration
FITC: Fluorescein isothiocyanate
HCS: High Content Screening
HDAC: histone deacetylase
HDACi: histone deacetylase inhibitor
HIC: hydrophobic interaction chromatography
HEPA: high efficiency particulate air
HPLC: high performance liquid chromatography
HRP: horseradish peroxidase
HSP: heat shock protein
IC_50_: half maximal inhibitory concentration
IHC: immunohistochemistry
MALDI: Matrix Assisted Laser Desorption Ionization
MST: median survival time
mAbs: monoclonal antibodies
NEAA: non-essential amino acids
PE: phycoerythrin
PBS: phosphate-buffered saline
PDX: patient-derived xenograft
RU: Response Units
SCID: Severe combined Immunodeficient
SDS-PAGE: Sodium Dodecyl Sulfate—PolyAcrylamide Gel Electrophoresis
PARP: Poly[ADP-ribose] polymerases
S.I.: International System
SPR: surface plasmon resonance
TCEP: tris[2-carboxyethyl]phosphine
TFA: *Trifluoroacetic acid*
UKCCR: United Kingdom Co-ordinating Committee on Cancer Research
UV: ultraviolet
VEGF: vascular endothelial growth factor.
