# Supplementary material for: ErbB2 Targeted Epigenetic Modulation: Anti-tumor Efficacy of the ADC Trastuzumab-HDACi ST8176AA1
Source: Front Oncol. 2020 Jan 23;9:1534. doi: 10.3389/fonc.2019.01534 (PMC6989603; doi:10.3389/fonc.2019.01534)

Supplementary Material

1 Supplementary Figures and Tables

1.1 Supplementary Figures

- **S1**: Scheme of synthesis of ADCs (conjugation of payloads to antibodies).
- **S2**: HIC chromatograms at 280 nm of naked trastuzumab and ADC ST8176AA1.
- **S3**: Native mass spectra obtained for trastuzumab and ADC ST8176AA1.
- **S4**: Deconvoluted mass spectrum of ADC ST8176AA1.
- **S5:** MALDI mass spectra of naked trastuzumab and ADC ST8178AA1.
- **S6:** Internalization of ST8176AA1 by tumor cells.
- **S7:** ST8176AA1 upregulates acetylation of histones and α-tubulin in tumor cells.
- **S8:** ST8176AA1 induces reversal of epithelial/mesenchymal transition in LS174T tumor cells.
- **S9:** ST8176AA1 induces the expression of ERα and ErbB2 in ErbB2+ and triple negative breast cancer cells.
- **S10**: Binding of trastuzumab to human breast cancer cell lines by cytofluorimetry.

Supplementary Figure 1.

The synthesis of antibody-drug conjugates (ADCs) ST8176AA1 and ST8178AA1 by conjugating the “payloads” ST8152AA1 and ST8128AA1 (both containing the HDACi ST7464AA1) to cysteine and lysine, respectively, of trastuzumab.

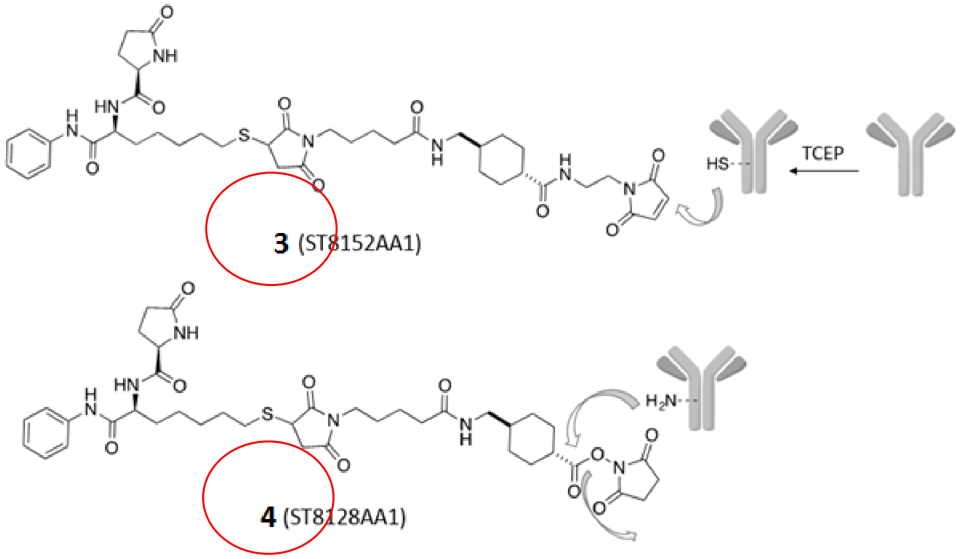

Supplementary Figure 2.

HIC chromatograms at 280 nm of naked trastuzumab *(top*) and ADC ST8176AA1 (DAR ~ 5.0) (*bottom*)

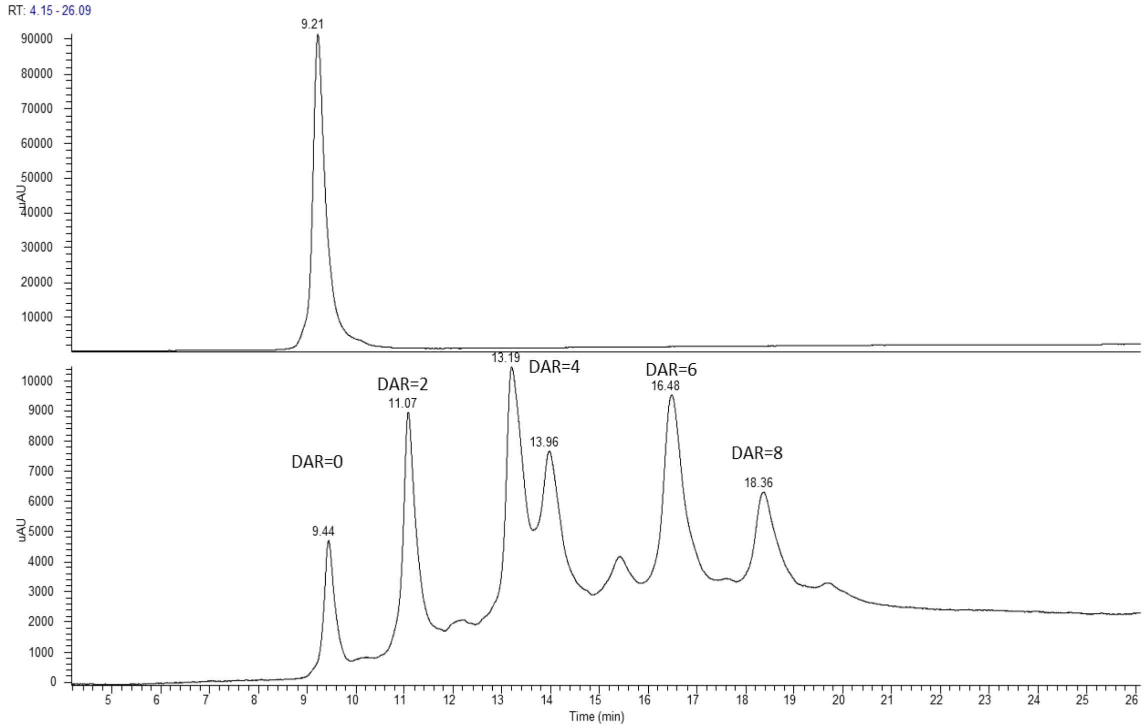

2

The average DAR was calculated considering the area of the peaks shown in the chromatograms and using the following formula, and in the reported case it was 4.6:

$$\overline{DAR} = \frac{\sum_{0}^{8} nA_{DAR_n}}{\sum_{0}^{8} A_{DAR_n}}$$

The DAR was measured also by SEC-MS under native conditions, performed on an UHPLC Ultimate 3000 (Thermo Fisher Scientific) using a column MabPac SEC-1 100 x 2.1 mm, 5µm (Thermo Fisher Scientific). The mobile phase was 50 mM ammonium acetate pH 6.8. The flow rate was 70 µL/min, the column oven was hold at 25°C and 5 or 10 µL of each sample were injected into the column. The UHPLC was interfaced with a Q-Exactive mass spectrometer (Thermo Fisher Scientific) equipped with ESI ion source. The mass spectra were acquired at resolution 17.5 K and 35 K in the *m/z* range ranging between 4000 and 8000.

Supplementary Figure 3.

The mass spectra of ADC ST8176AA1 and Trastuzumab were reported. After deconvolution, the major component was attributable to the species containing four drugs and the calculated DAR was 4.12, as shown on the left side of the Figure S4.

Native mass spectra obtained for Trastuzumab (*bottom*) and ADC ST8176AA1 (*top*).

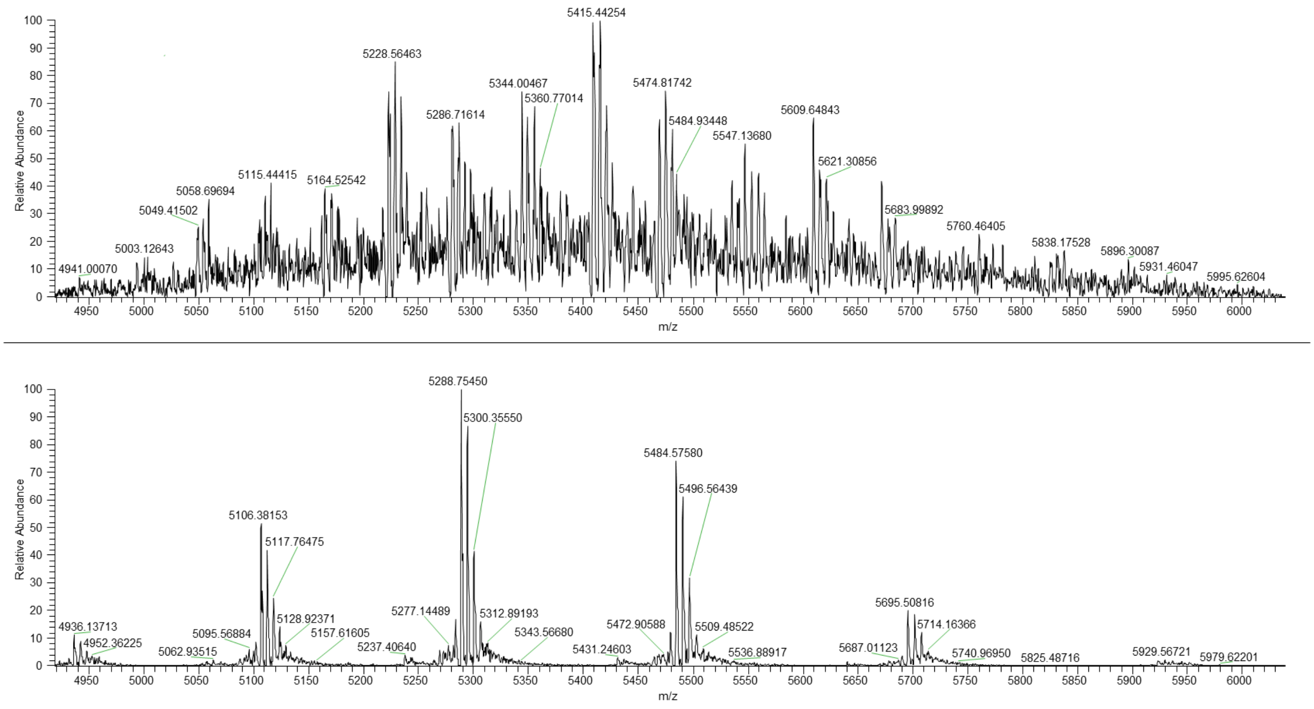

Supplementary Figure 4.

Deconvoluted mass spectrum of ADC ST8176AA1.

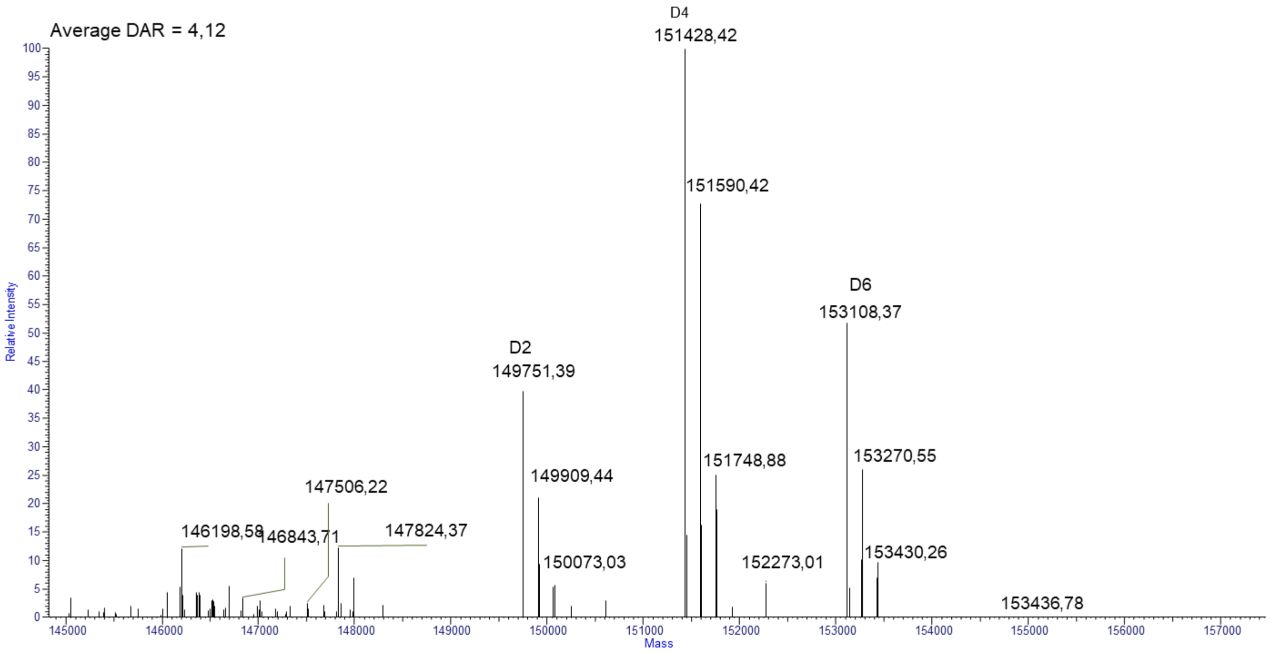

Supplementary Figure 5.

MALDI mass spectra of naked Trastuzumab, with M.W. ~ 147 kDa (*top*) and ADC ST8178AA1, with M.W. ~ 153 kDa – DAR~ 8 (*bottom*).

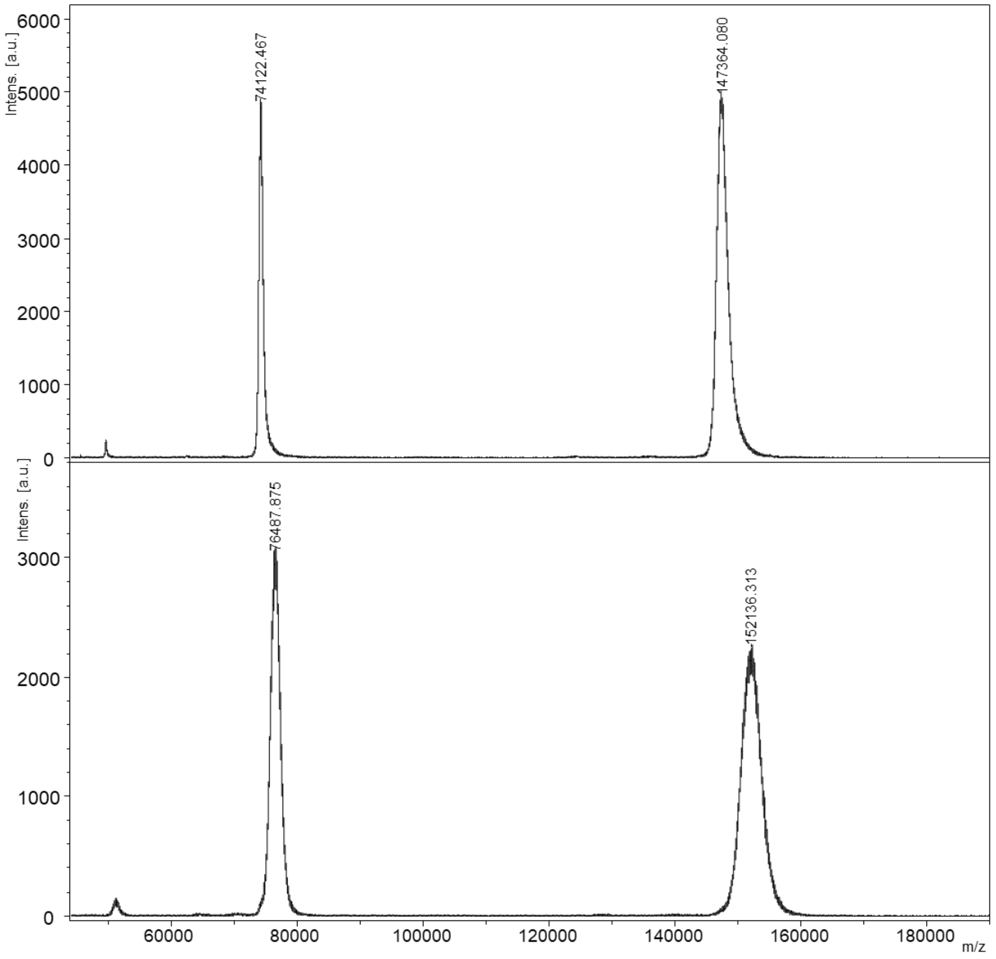

Supplementary Figure 6.

Internalization of ST8176AA1 by tumor cells. Internalization of ST8176AA1 and trastuzumab (both at 5 µg/mL) by LS174T (colon) and N87 (stomach) human carcinoma cells, as measured by HCS fluorescence imaging after 1 hour incubation. After washing, the cells were fixed and stained by using FITC-conjugated mouse anti-human Ig (blue signal). Draq5 dye staining of nucleus (grey). Each image is representative of at least 5 fields of duplicate wells. Magnification 60X. Data are from one representative experiment out of two.

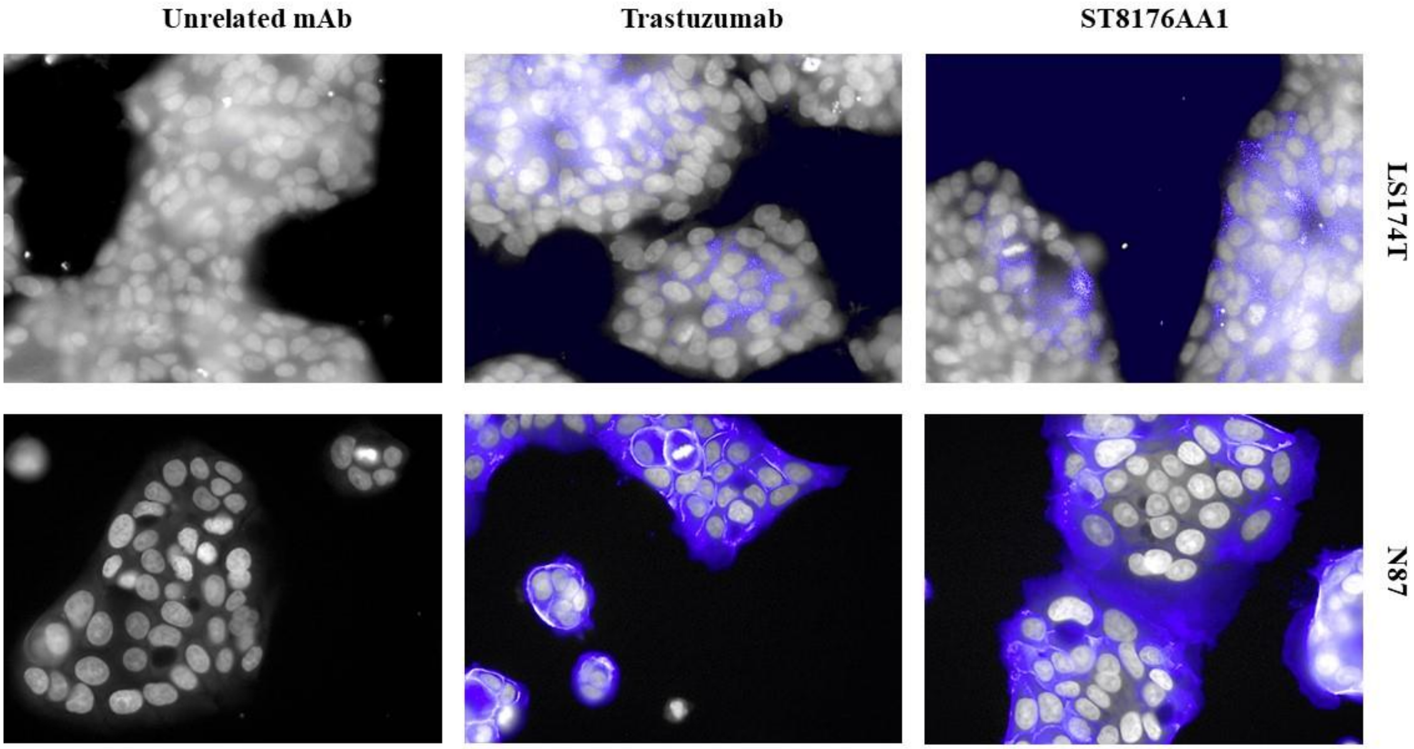

Supplementary Figure 7.

ST8176AA1 upregulates acetylation of histones and alpha-tubulin in tumor cells. HCS imaging showing acetylation of alpha-tubulin (red) and histone H3 (fuchsia) in SKBR3 (breast) and SKOV3 (ovary) carcinoma cells. ST8176AA1 and trastuzumab were used at 5 µg/mL (3 hour incubation). All panels, insets show specific fluorescence signals within the cells. Draq5 dye staining of nucleus (grey). Each image is representative of at least 5 fields of duplicate wells. Magnification 60X. Data are from one representative experiment out of two.

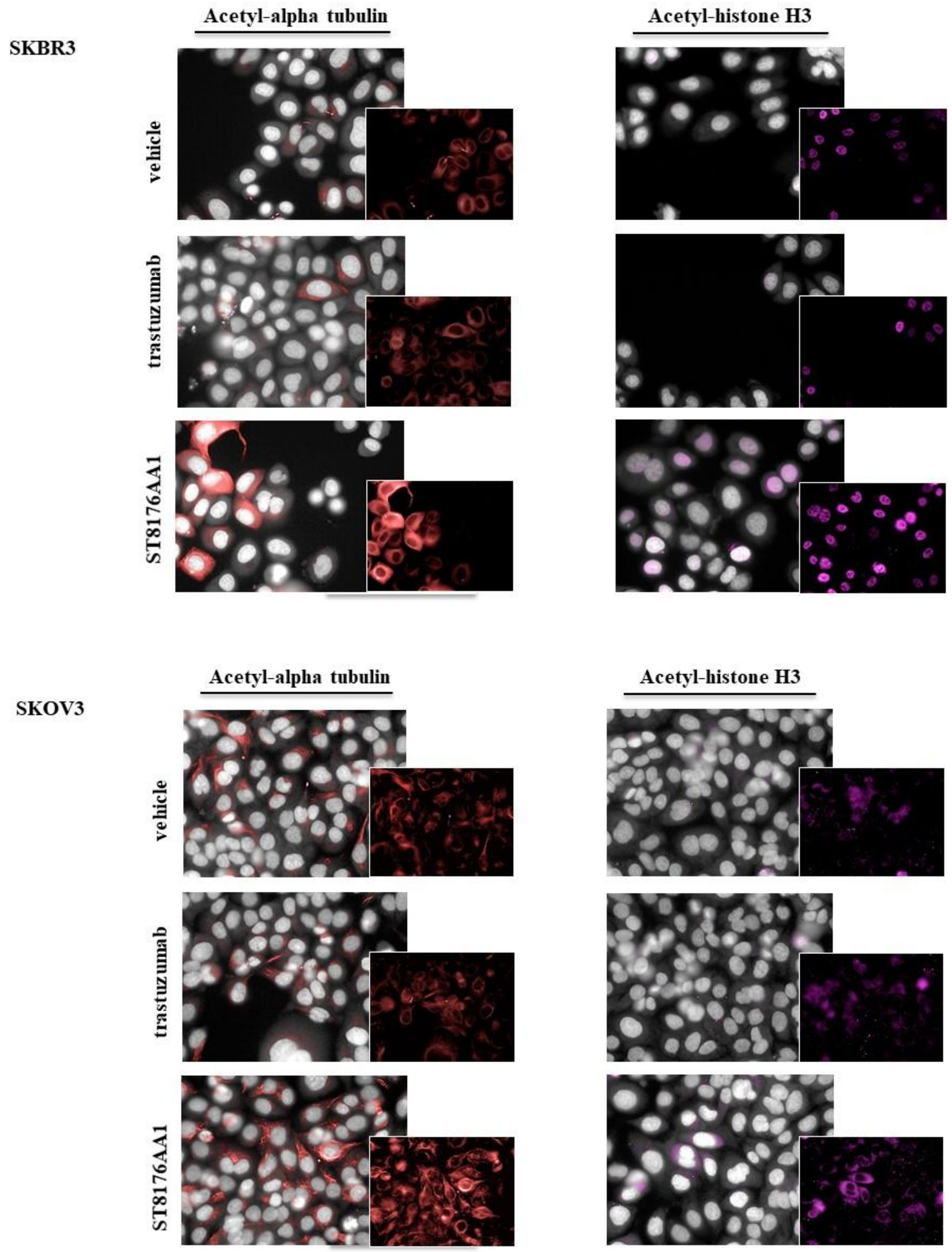

Supplementary Figure 8.

ST8176AA1 induces reversal of epithelial/mesenchymal transition in LS174T tumor cells. HCS imaging analysis showing that LS174T cells treated with ST8176AA1 exhibit increased expression of the claudin2 and E-cadherin (epithelial) proteins and reduction of vimentin and fibronectin (mesenchymal) proteins. ST8176AA1 and trastuzumab were used at 15 µg/mL (3 day incubation). In all panels, insets show specific fluorescence signals within the cells. Draq5 dye staining of nucleus (grey). Each image is representative of at least 5 fields of duplicate wells. Magnification 60X. Data are from one representative experiment out of two.

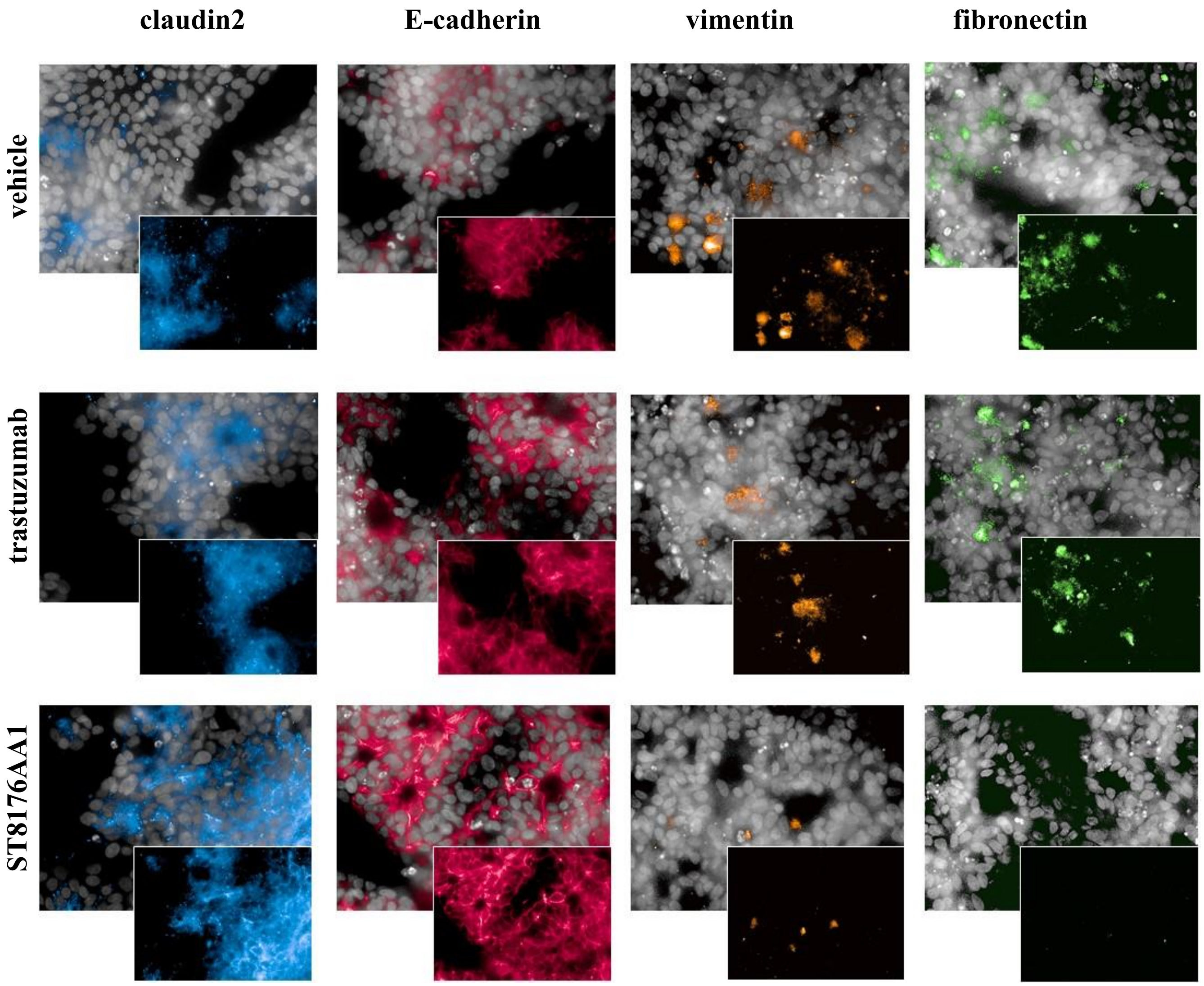

Supplementary Figure 9.

ST8176AA1 induces the expression of ERα and ErbB2 in ErbB2+ and triple negative breast cancer cells. HCS fluorescence imaging analysis show that ErbB2+ (MCF7) and triple negative (MDA-MB231) breast cancer cells treated with ST8176AA1 exhibit increased expression of ERα (purple) and ErbB2 (green) proteins, compared to trastuzumab. ST8176AA1 and trastuzumab were used at 5 µg/mL (3 day incubation). Basal ErbB2 expression scored by cytofluorimetry in brackets. In all panels, insets show specific fluorescence signals within the cells. Draq5 dye staining of nucleus (grey). Each image is representative of at least 5 fields of duplicate wells. Magnification 60X. Data are from one representative experiment out of two.

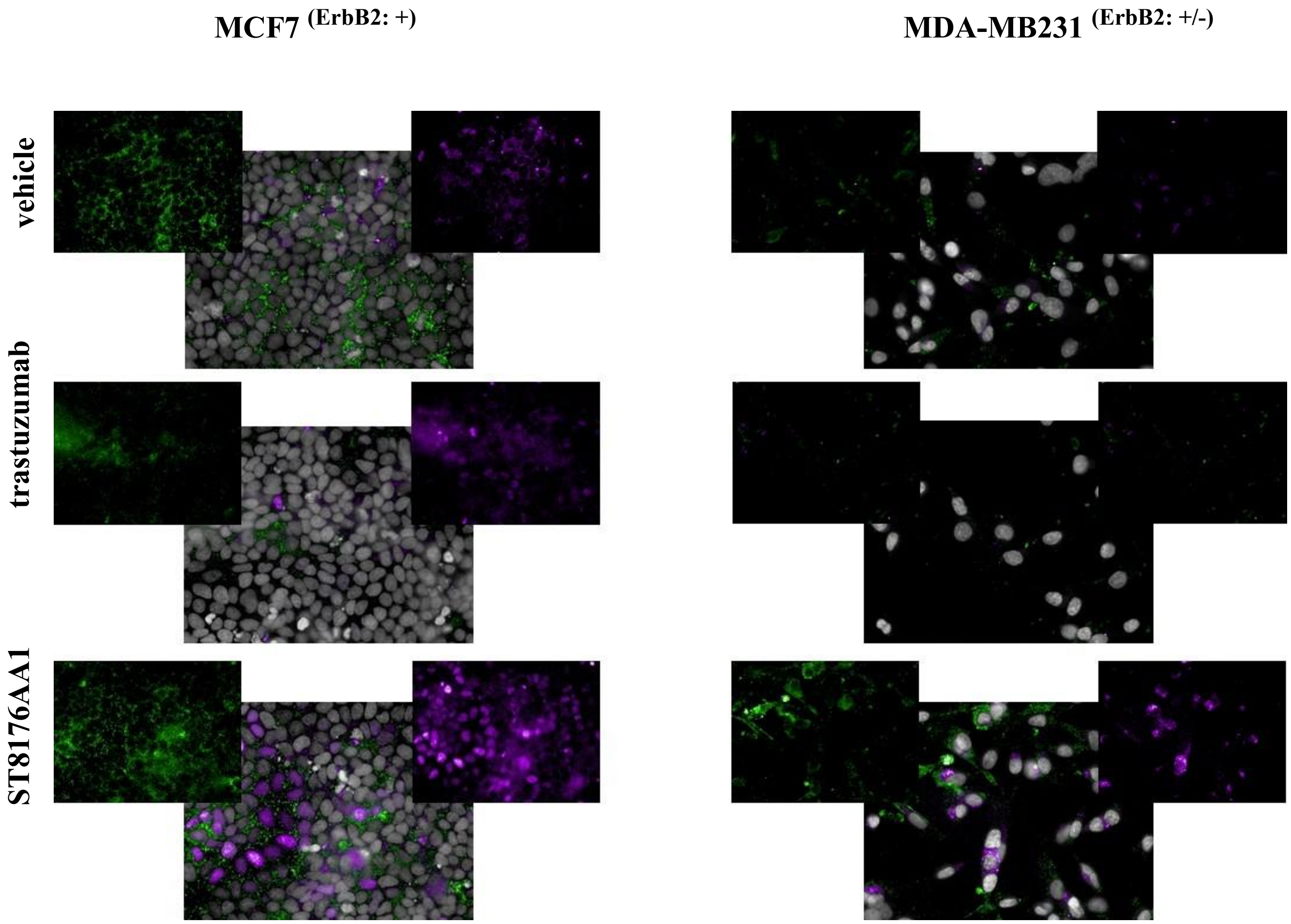

Supplementary Figure 10.

Binding of trastuzumab to human breast cancer cell lines by cytofluorimetry. Cells were incubated 1 hour in ice with trastuzumab, washed two times with PBS and then incubated with FITC-conjugated mouse anti-human Ig (Becton Dickinson). Cells were analyzed by FACScalibur (Becton Dickinson). Black peaks refer to cells without primary antibody. Analysis was performed on propidium iodide-negative cells.

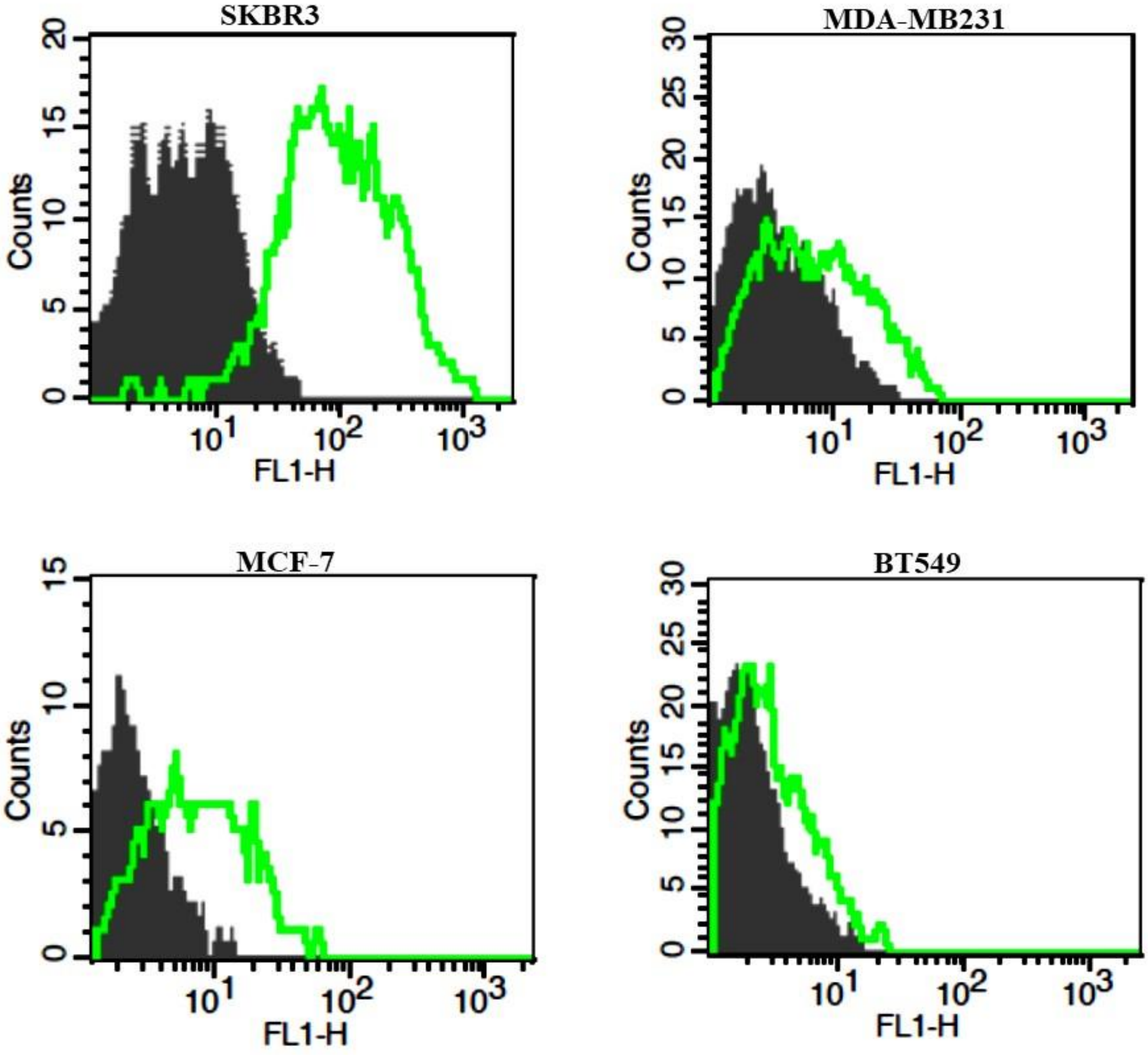

Supplement: Supplementary file 1 [file Data_Sheet_1.PDF]
